# Interdisciplinary fetal-neonatal neurology training applies neural exposome perspectives to neurology principles and practice

**DOI:** 10.3389/fneur.2023.1321674

**Published:** 2024-01-15

**Authors:** Mark S. Scher

**Affiliations:** Division of Pediatric Neurology, Department of Pediatrics, Case Western Reserve University School of Medicine, Cleveland, OH, United States

**Keywords:** fetal-neonatal neurology, neural exposome, reproductive health, pregnancy, social determinants of health, toxic stressors, exosomes, developmental origins

## Abstract

An interdisciplinary fetal-neonatal neurology (FNN) program over the first 1,000 days teaches perspectives of the neural exposome that are applicable across the life span. This curriculum strengthens neonatal neurocritical care, pediatric, and adult neurology training objectives. Teaching at maternal-pediatric hospital centers optimally merges reproductive, pregnancy, and pediatric approaches to healthcare. Phenotype–genotype expressions of health or disease pathways represent a dynamic neural exposome over developmental time. The science of uncertainty applied to FNN training re-enforces the importance of shared clinical decisions that minimize bias and reduce cognitive errors. Trainees select mentoring committee participants that will maximize their learning experiences. Standardized questions and oral presentations monitor educational progress. Master or doctoral defense preparation and competitive research funding can be goals for specific individuals. FNN principles applied to practice offer an understanding of gene–environment interactions that recognizes the effects of reproductive health on the maternal-placental-fetal triad, neonate, child, and adult. Pre-conception and prenatal adversities potentially diminish life-course brain health. Endogenous and exogenous toxic stressor interplay (TSI) alters the neural exposome through maladaptive developmental neuroplasticity. Developmental disorders and epilepsy are primarily expressed during the first 1,000 days. Communicable and noncommunicable illnesses continue to interact with the neural exposome to express diverse neurologic disorders across the lifespan, particularly during the critical/sensitive time periods of adolescence and reproductive senescence. Anomalous or destructive fetal neuropathologic lesions change clinical expressions across this developmental-aging continuum. An integrated understanding of reproductive, pregnancy, placental, neonatal, childhood, and adult exposome effects offers a life-course perspective of the neural exposome. Exosome research promises improved disease monitoring and drug delivery starting during pregnancy. Developmental origins of health and disease principles applied to FNN practice anticipate neurologic diagnoses with interventions that can benefit successive generations. Addressing health care disparities in the Global South and high-income country medical deserts require constructive dialogue among stakeholders to achieve medical equity. Population health policies require a brain capital strategy that reduces the global burden of neurologic diseases by applying FNN principles and practice. This integrative neurologic care approach will prolong survival with an improved quality of life for persons across the lifespan confronted with neurological disorders.

## Introduction: the neural exposome concept applied to fetal neonatal neurology training

Neuroscience research regarding the influence of toxic stressor interplay (TSI) on the neural exposome promotes diagnostic and therapeutic strategies that will more effectively reduce adverse outcomes across the lifespan. Interdisciplinary fetal-neonatal neurology (FNN) training contributes to this effort. An FNN program encompassing the first 1,000 days has previously been described that teaches analytic skills that will enhance career-long learning (1). These educational perspectives strengthen both pediatric and adult neurology training objectives that will improve health care (2).

Current pediatric neurology training programs do not effectively offer a comprehensive knowledge base in FNN for career-long learning. A past survey of pediatric subspecialists ranked pediatric neurologists as the least likely to have received formal FNN training and were the least prepared to participate in prenatal pediatric evaluations (3). An expanded interdisciplinary curriculum would strengthen the trainee’s FNN principles to be applied to practice. Improved diagnostic approaches would offer more effective neurotherapeutic interventions for persons with neurologic disorders.

The United Council for Neurologic Subspecialties (UCNS) in the United States recently approved an accredited one-year program in neonatal neurocritical care. This subspecialty training experience helps close the educational gap with a greater emphasis on neonatal rather than fetal neurology topics. This program emphasizes diagnostic and therapeutic interventions for the symptomatic neonatal minority who primarily express encephalopathy, seizures, stroke, and brain disorders of prematurity.

These phenotypes, however, often represent a continuum of complex prenatal-to-neonatal disease pathways. Reproductive and pregnancy-related illnesses can contribute to fetal brain lesions within the maternal-placental-fetal (MPF) triad and/or increase risks for peripartum brain injuries. Anomalous and destructive brain lesions may have occurred during pregnancy that influenced phenotypic presentations during fetal to neonatal transitions.

Prenatal disease pathways are often undetectable or lack diagnostic clarity given the sensitivity and specificity limitations of current fetal surveillance biomarkers. FNN training strengthens neonatal neurocritical healthcare delivery by integrating maternal with pediatric healthcare priorities. Prenatal perspectives help anticipate MPF triad susceptibilities to diseases expressed during peripartum and neonatal life. Future obstetrical and neonatal intensive care interventions will offer more effective strategies to improve outcomes for this symptomatic neonatal minority.

Pediatric neurologists primarily encounter children who experienced earlier prenatal brain disorders without abnormal clinical presentations. FNN training provides a prenatal diagnostic frame of reference to consider preclinical disease pathways when evaluating this “silent majority” for suspected neurologic diseases (1). Participation in fetal neurology consultations provides opportunities to consider diagnoses during pregnancy that support childhood evaluations when neurologic disorders present.

**Figure 1:**
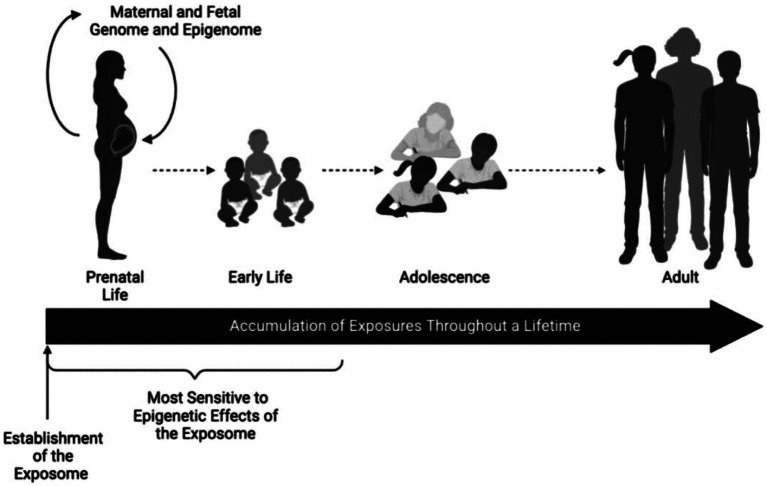
The epigenome remains sensitive to environmental insults. Epigenetic patterns are established before conception and maintained within rapidly developing tissues during pregnancy and early postnatal life. These environmental perturbations to epigenetic programming remain stable and can serve as biomarkers of past exposures or predictors of future disease later in the life course. Exposures during childhood, adolescence, and adulthood may impart additional epigenetic alterations. Reprinted with permission Colwell et al. (4) licensed under CC by 4.0.

Neurology residents benefit from FNN training when evaluating children through adolescence. Integration of FNN principles into practice by pediatric and adult neurologists improves diagnostic accuracy across the lifespan relative to communicable and non-communicable diseases that further diminish brain health. Considerations of reproductive, pregnancy, and placental exposomes influence evaluations of MPF triads, neonates, children, and adults. Diseases and adversities throughout the lifetime are represented by a dynamic neural exposome (4) (Figure 1). This life-course perspective will require novel multimodal biomarkers to select more effective preventive, rescue, and reparative neuroprotective interventions with advancing ages.

## A dual diagnostic approach applies the neural exposome concept

Advances in developmental neurotoxicology help improve pediatric neurology principles and practice (5). Pediatric neurology training benefits from an understanding of the neural exposome integrated with environmental neuroscience. Neurologic disease onset and expression during the first 1,000 days reflect etiopathogenetic pathways associated with toxic stressor interplay (TSI). Interactions among multiple endogenous and exogenous toxic stressors negatively influence a dynamic neural exposome across childhood into adulthood to diminish brain health (6).

A two-step diagnostic strategy helps promote person-centered decisions by considering TSI: 1-time-dependent phenotypic descriptions identify diseases that affect each woman, MPF triad, neonate, and child relevant to the specific maturational stage of the child’s nervous system; 2-etiopathogenetic pathways consider TSI during each developmental niche to identify the predominant disease mechanisms expressed by a particular phenotype. Serial assessments reconsider dynamic gene–environment (G × E) interactions over the first 1,000 days, initially influenced by a woman’s reproductive health (1). This first critical/sensitive period of developmental neuroplasticity contributes to 80% of neuronal connectivity by 2 years of age (7). Neuroadaptive effects of TSI later continue, particularly during the critical/sensitive periods of adolescence and reproductive senescence to express life-long brain health or disease.

Knowledge of a woman’s health before conception facilitates assessments of disease risks of the MPF triad that influence fetal brain development during each pregnancy. Developmental neuroplasticity principles emphasize periconceptional effects on first-trimester embryonic and fetal brain structures. Progenitor neuronal/glial populations within transient brain regions determine subsequent fetal brain maturation across three trimesters. Positive adaptative interactions by the MPF triad preserve normal fetal brain functions. Negative adaptations alternatively contribute to contemporaneous or delayed expressions of fetal brain disorders. Abnormal clinical signs during the transition from fetal to neonatal life may require neonatal neurointensive care for a minority of all children who later express neurologic sequelae.

A “silent majority” of healthy neonates often without detectable prenatal diseases more likely will present with childhood neurologic disorders. FNN training experiences consequently require a pediatric component to address or re-assess diagnostic decisions of children during the first 2 years of life. Primary care providers’ wellness strategies (8) request early intervention referrals to an FNN program when neurologic concerns are suspected, sometimes challenged by adverse childhood effects based on healthcare disparities or suboptimal parenting (9, 10). Time-sensitive pediatric subspecialty consultations also contribute to neurology referrals when multi-systemic communicable and non-communicable diseases are encountered during outpatient and hospital evaluations. Pediatric intensive care assessments focus on the most severely affected children, many of whom were survivors after requiring neonatal neurocritical care.

Pediatric neurologists serving children through adolescence continue to apply FNN principles to practice by merging developmental origins and life-course concepts to provide effective medical and educational interventions. School performance is monitored by the pediatric neurologist with the family’s and school’s attention to the child’s neurologic and mental health. Educational neuroscience approaches consider knowledge of the neural exposome to optimally apply effective lesson plans (11–13). Mainstreamed students who express functional neurodiversity are more likely to adapt positively to teaching strategies. Those children with more challenging developmental disorders benefit from person-centered individual educational plans that also provide physical, occupational, speech-language, vision, and feeding therapies (14).

Reproductive, prenatal, and early life adversities also contribute to diverse mental health disorders that present across the lifespan. Pediatric neurologists often consult with pediatric psychologists and psychiatrists regarding children who express behavioral phenotypes that require mental health interventions.

Mental health care providers can benefit from the application of FNN principles to their practices. More effective diagnostic and therapeutic decisions are formulated for a range of disorders from anxiety, attention/hyperactivity, depression, and mood disorders to autistic spectrum disorders and thought disorders. The updated Diagnostic and Statistical Manual of Mental Disorders (DSM-5 TR), for example, suggested revisions to the previously published DSM, inclusive of children and adults expressing a broader range of autistic behaviors that challenge diagnostic accuracy (15).

Long intervals often separate disease onset from phenotypic expression (16). Intervening maladaptive G x E interactions therefore require serial evaluations to anticipate preclinical neurologic disorders or detect ambiguous early clinical signs using available testing tools. Structural-functional reassessments across developmental-aging time periods are therefore required, starting during pregnancy. Limitations in clinical decision-making must apply the science of uncertainty (17), given the inability to apply hindsight analyses in real time. Metadata analyses utilizing deep learning strategies will help enhance diagnostic and therapeutic approaches, applicable to person-centered shared decisions among all stakeholders. Innovative strategies will advance diagnostic skills using FNN perspectives.

The cognitive biases of providers and families diminish accuracy with each clinical encounter. Strategies to minimize errors of omission or commission have been extensively discussed (18). Dual cognitive processing using a “fast thinking-slow thinking” diagnostic approach was presented almost 50 years ago (19) with interdisciplinary applications. This strategy applied to healthcare helps preserve accuracy, cooperation, and trust among all stakeholders (17, 20). Re-evaluations address ongoing potential biases during critical-sensitive periods of neuroplasticity when changes in brain structure and function reflect diverse clinical expressions of a dynamic neural exposome.

Biosocial principles are important components of FNN training, applied to shared clinical decisions to reduce or avoid bias. Bioethical discussions begin with reproductive and pregnancy healthcare practices, particularly given worldwide legislative and judicial decisions that have reduced a woman’s reproductive rights (21, 22) that affect a child’s brain health. Parents of profoundly delayed children especially require support and partnerships that prioritize bioethical decisions. Interdisciplinary collaborations exemplified by the ALIGN program have recently been applied to neuropalliative care to promote effective shared clinical decisions (23). This approach is relevant to all diagnostic challenges.

**Figure 2:**
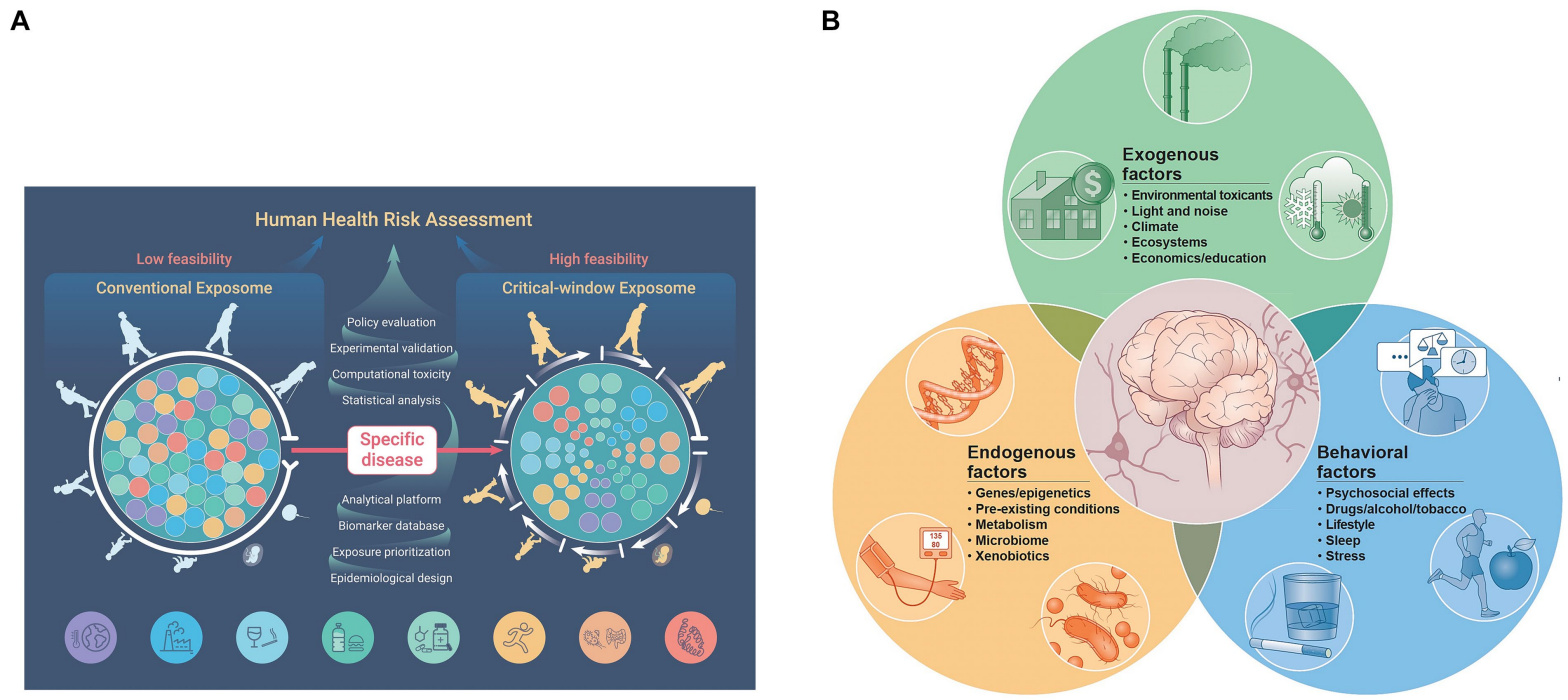
**(A)** The functional exposome prioritizes person-centered exposomic factors based on the developmental-aging continuum from conception to old age. This approach improves feasibility from conventional to critical window exposome applications to better assess human health risk (note the color order representing each person). Critical-sensitive windows correlate specific disease expressions within each developmental-aging stage across the lifespan. Exogenous and endogenous toxic interplay responsible for the specific disease is meta-analyzed and prioritized using large data sets applied to person-centered metrics. This can be studied by the use of appropriate epidemiological design, followed by exposure prioritization, biomarker databases, analytical platforms, statistical analyses, computational toxicity estimations, experimental validation, and health policy applications, standardized to a specific country or region. Reprinted with permission Fang et al. (24) licensed under CC by 4.0. **(B)** The neural exposome is depicted as overlapping influences between multiple endogenous and exogenous toxic stressors. Reprinted with permission Tamiz et al. (25) licensed under CC by 4.0. Note that endogenous factors also include mental health diseases combined with social determinants of health and lifestyle choices. Examples are listed that also include a more extensive list of factors.

The neural exposome concept (24, 25) integrates environmental neuroscience into life-course neurologic diagnostic strategies (26) (Figures 2A,B). FNN training offers analytic skills to anticipate age-dependent expressions of the neural exposome. TSI involving numerous endogenous and exogenous factors potentially diminishes the brain health of susceptible mother–child dyads. Debiasing strategies help re-interpret challenging historical, examination, and test results across developmental time. Applying the science of uncertainty to FNN practice helps consider complexities of TSI when offering neurologic diagnoses (27).

FNN training promotes effective interventions throughout the lifespan, beginning during the first 1,000 days when children first express developmental/behavioral disorders and epilepsies. Neurologic and mental health disorders present during later childhood and adolescence, requiring reassessments to maintain diagnostic accuracy. Preconception and pregnancy experiences contribute to adult disease expressions (28). Neurologic and mental health disorders expressed by older persons also require FNN perspectives. Effective interventions can help lengthen survival with an improved quality of life.

Social determinants of health strongly influence outcomes. Equitable healthcare delivery is required for all women, MPF triads, children, and adults experiencing neurologic disorders (14). These strategies help sustain brain health across generations in response to TSI effects on the neural exposome. Addressing modifiable factors using effective neurotherapeutic interventions must initially recognize medical equity regardless of race, ethnicity, economic status, gender identity, and geographic isolation (29).

## Organization and workflow for the FNN trainee

FNN trainees would more optimally benefit from clinical and didactic interdisciplinary instruction pertaining to the first 1,000 days for a suggested two-year experience to accommodate an expanded curriculum (Appendices 1, 2). This training would extend the one-year fellowship currently offered by neonatal neurocritical care programs. This additional time would reinforce diagnostic, therapeutic, and prognostic accuracy applicable to the four “great neonatal neurological syndromes” (1) (Table 1).

**Table 1:** Clinical opportunities for FNN training.

Clinical venues	Training content
Preconception surveillance Obstetrical service Pediatric service	Maternal-pediatric diseases/adverse conditions High risk pregnancy planning-complex diagnoses Adolescent pregnancy-associated risks
First trimester surveillance Maternal levels of care Sonography Genetic/serologic screening	Planned versus unplanned pregnancies Placental implantation abnormalities Aneuploidy, wes, gwas results Communicable/non-communicable disease screening
Second trimester surveillance Maternal level of care Anatomic survey MFM referrals	Neuroaxis lesions
	Multisystemic diseases of the maternal-placental-fetal triad
	Primary brain anomalous/destructive lesions
	Secondary brain effects from systemic illnesses
MFM interdisciplinary service Fetal neurology consultations MFM conferences Fetal imaging service	Serial assessments of disease conditions
	Sonographic anthropometric, fetal MRI findings Brain, placenta, specific organ-systems
	Maternal infections, hypertension, metabolic diseases, mental health Maternal ICU care
Third trimester surveillance	Reasons for maternal admission to deliver/intrapartum monitoring findings
Maternal hospitalizations	
Peripartum/neonatal services Transitional/acute stage Subacute-stepdown care Convalescent care-discharge planning	Resuscitation/early ICU stabilization/ medical interventions
	Organ-system specific requiring pediatric services-nursing interventions-therapy services
	Health maintenance/wellness programs
	Referrals for developmental risk/ the medically fragile child
Pediatric services Primary care referrals Intervention services Neonatal follow up program Outpatient pediatric subspecialty referrals Inpatient pediatric referrals Emergency room Pediatric intensive care Epilepsy service Sleep service	System-specific surveillance
	Acute disease presentations

Reproductive and pregnancy health perspectives would be introduced to trainees through participation in fetal neurology consultations. These experiences are best offered at maternal and pediatric hospitals that are in proximity, where obstetrical, maternal-fetal medicine and genetic services can more efficiently coordinate fetal neurology referrals. Evaluations may originate in the outpatient maternal care setting, the maternal imaging center, high risk interdisciplinary MFM conferences, and during hospitalizations for pregnant women, including those requiring intensive care. Maternal reportage of relevant signs and symptoms guides these evaluations. Familiarity with trimester-specific obstetrical diagnostic pathways requires instruction in the interpretation of serial fetal surveillance test results. Considerations include serologic, genetic, and proteomic biomarkers using different body fluid analyses, compared with fetal anthropometric measures, biophysical scales, and doppler flow patterns using sonography.

Peripartum management strategies offer an understanding of the challenges involved in the choice of an optimal obstetrical clinical pathway during a problematic labor and/or delivery. Interpretative uses and limitations of intrapartum fetal heart rate patterns, intrauterine pressure monitors, and fetal scalp oximetry results need to be discussed. Resuscitation and triage procedures requiring neonatal intensive care are essential skills to address multisystemic priorities for neonatal survival and stabilization during the first crucial hours of adaptation from fetal to neonatal life (30).

Neonatal neurocritical care highlights interventions for encephalopathy, seizures, brain disorders of prematurity, and stroke. These conditions are integrated into a multi-system approach during daily work rounds that continue from acute through stepdown stages of care until discharge. The FNN trainee will incorporate learning experiences from both prenatal and neonatal consultations, strengthened by didactic tutorials by faculty from multiple pediatric subspecialties.

An FNN program requires a comprehensive pediatric follow-up program. Consultations with primary pediatric and subspecialists address neurologic clinical presentations through the child’s second birthday. This curriculum will also strengthen pediatric and adult neurology residents’ diagnostic perspectives.

Interdisciplinary FNN perspectives will be augmented by faculty representing neonatology, obstetrics, pathology, neuroradiology, developmental-behavioral pediatrics, genetics, nursing, and therapy approaches. Pediatric subspecialties provide greater information regarding system-specific health and disease. Faculty representing computer science-engineering, bioethics, health policy, epidemiology/statistics, population health, health-system design, and family advocacy further reinforce interdisciplinary training synergy. Current peer-reviewed literature will be discussed at regularly scheduled didactic sessions, applied during each clinical encounter (Appendices 1, 2). Electives at selected institutions will add to the trainee’s learning experiences with faculty who have advanced skills such as neuroimaging, neurophysiology, and neurogenetics.

Trainees will participate in their mentoring committee selection comprised of participants who best support their career paths. Research projects are designed with committee recommendations for manuscript submission based on a chosen topic relevant to the trainee’s interest. Preparation for a master’s or doctoral defense as well as applications for competitive research funding will apply to designated trainees. Examinations will assess FNN practice competency using approved questions by recognized professional educational organizations such as the American Accreditation of Graduate Medical Education in the United States and the United Council of Neurologic Subspecialties in the United States.

Interrelationships among reproductive, pregnancy, placental, neonatal, childhood, and adult exposomes are now discussed that are relevant to neurology principles and practice, emphasizing FNN through the curriculum proposals discussed above. This review concludes with suggestions for new neurotherapeutic approaches that will support a capital brain capital health strategy across the lifespan.

## The reproductive health exposome

Girls and women of reproductive age are vulnerable to adverse exposomic effects prior to each pregnancy. Health choices and experiences during the periconceptional period more proximately influence pregnancy health. Medical risks are increased with unplanned pregnancies (31), particularly for adolescents (32, 33). TSI continues to threaten MPF triad health during each pregnancy. A social exposome introduces vulnerabilities to individuals and populations who experience health disparities, resulting in reduced health outcomes across all stages of life (34).

System-specific diseases experienced by the girl or woman such as hypertension, diabetes, obesity, and mental health disorders introduce TSI which potentially impairs her reproductive health. These preconception adverse effects amplify diseases during pregnancy affecting the MPF triad. Many disorders such as polycystic ovarian syndrome and genetic syndromes are particularly challenging given the multi-systemic clinical effects from TSI. Xenobiotic exposures from thousands of pollutants combined with social determinants of health and lifestyle choices represent the complexity of exogenous toxic stressors. These exposures interact with each woman’s endogenous biological factors, representing preclinical or active disease states that contribute to brain lesions of her fetus.

Exaggerated inflammatory, oxidative, and hypoxic–ischemic reproductive disease pathways are etiopathogenetic mechanisms associated with TSI that introduce adversities to the MPF triad. “Omics” science using deep learning technologies will provide optimal testing for planned pregnancies. Periconceptional and first-trimester biomarkers will better detect and monitor pregnancy-related diseases (35, 36). Maternal-fetal medicine consultations may be requested to enhance surveillance to provide time-sensitive interventions.

Allelic abnormalities in the parental gametes or *de novo* genetic defects produced by the conceptus may subsequently worsen through post-translational disease processes, contributing to the MPF triad’s disease burden. Future whole exome sequencing and targeted high through-put next-generation genomic technologies before and following conception will more likely determine inherited genetic risks despite negative karyotypic and microarray screens (37, 38). Future epigenetic biomarkers such as the methylome will assess biological maladaptation with increasing gestational ages (39, 40) as diseases or adversities present or worsen during pregnancy. These post-translational diagnostic tools will help expand the detection of prenatal G × E interactions (41) to more accurately predict neurologic risks across the lifespan (42). Impaired fetal brain development experienced during an earlier pregnancy may re-occur during subsequent pregnancies based on transgenerational risk through epigenetic memory (43).

## The pregnancy exposome

Developmental neuroscience principles are relevant to an understanding of the neural exposome and guide FNN training objectives during each pregnancy. No current clinical biomarkers comprehensively assess all relevant factors that influence the woman’s pregnancy exposome. Maternal levels of care such as those applied in the United States rely on a health provider’s choice of fetal surveillance testing procedures (44). Present diagnostic choices using these tests have limited specificity and sensitivity to detect most fetal brain disorders.

Teaching environmental science to obstetrical/maternal-fetal medicine trainees and providers is now advocated. Improvements in maternal health by reducing TSI will have life-course benefits for women and children (45). Environmental threats in the Global South (i.e., countries in the regions of Latin America, Africa, Asia, and Oceania) and high-income country medical deserts (HICMD) (46) present greater challenges for healthcare delivery, given a paucity of resources for these vulnerable populations according to recommended maternal levels of care (47).

Research methodologies are currently underpowered by small sample sizes, exclusion of vulnerable populations, and biased choices of study variables. Interdisciplinary FNN training can help close this information gap regarding TSI by combining the MPF triad with neonatal assessments. Study designs need to prioritize medical equity with person-centered evaluations before and during each pregnancy (48).

Physiologic adaptations by the pregnant woman are needed to preserve MPF triad health (49) since endogenous and exogenous TSI can potentially diminish health through adverse biological-chemical interactions involving inter-connected organ-systems. Hypertensive, metabolic, mental health, and autoimmune-inflammatory disease states exemplify periconceptional conditions encountered by women that may worsen during each pregnancy. TSI reduces the health of susceptible maternal-fetal dyads which can impair the neural exposome. Maternal body fluids, placental biomarkers, and cord blood samples offer opportunities to assess the maternal and fetal micro-environments (50). Few studies have evaluated TSI in pregnant women using biomarkers that specifically monitor risks for fetal brain disorders.

A more comprehensive investigation of the pregnancy exposome offers insights into the diagnosis of fetal brain disorders associated with trimester-specific TSI. Biomarkers representing a range of exposures will better detect biological-chemical interactions that contribute to maternal diseases (51). Representative noncommunicable and communicable maternal diseases and adversities are discussed below that highlight the importance of the pregnancy exposome.

### Hypertensive disorders

Chemical and biological interactions are expressed as hypertensive disorders of pregnancy (PIH), which affects 5–8% of the world’s female population (52). One preeclampsia study assessed placental samples using non-targeted measures (53). The report’s findings lay the foundation for future investigations of omics-based systems biology: (1) biological mechanisms/pathways linking gene signatures, human metabolism compounds, and physiological effects; (2) specific xenobiotics or human metabolites that may be identified as risk factors; and (3) exposure sources of xenobiotics (primary vs. secondary, intentional vs. unplanned) that were observed in individuals. Companion studies reinforce these findings by reporting that maternal levels of phthalates, perfluoroalkyl acids (54), and endocrine disruptors (55) contribute to placental dysplasia of women associated with PIH. Integrative exposomic, transcriptomic, and epigenomic analyses of human placental tissue can potentially link numerous understudied chemicals contributing to PIH (53).

### Gestational diabetes mellitus and diabesity

Under and over-nutrition during pregnancy together with exposure to environmental pollutants such as heavy metals, endocrine-disrupting chemicals, pesticides, drugs, pharmaceuticals, lifestyle, air pollutants, and tobacco smoke play important roles in the development of gestational diabetes mellitus (GDM). Metabolic stress from obesity increases the risk of GDM and comorbid diseases during and following each pregnancy. Clinical risk factors for GDM development require an understanding of disease pathways. Reducing the occurrence and consequences of GDM requires knowledge-based interventions that identify harmful effects of TSI (56) because of glucose intolerance. The negative impact on placental vasculature function with GDM and gestational diabesity has been reviewed (57). This will be reemphasized in the placental exposome section describing ischemic placental syndrome which contributes to two of multiple great obstetrical syndromes associated with PIH and GDM (58, 59).

### Autoimmune disorders

Non-infectious autoimmune disease pathways are associated with maternal inflammatory disorders. Interactions between environmental exposures and the MPF triad’s immune system play important roles in fetal, neonatal, or childhood brain disorders. Pregnancy presents a biologically unique physiological scenario during the woman’s reproductive years. Her immune system must adjust to maintain the health of the semi-allogenic fetus. Trimester-specific alterations in the balance between pro-and anti-inflammatory states facilitate uterine tissue remodeling, fetal growth, and development. Fetal tolerance must be maintained without compromising homeostatic maternal immune system functions to protect the maternal-fetal dyad throughout pregnancy (60).

Novel bioinformatics tools using systems immunology and machine learning are presently being studied by a large European consortium. Large datasets are being analyzed to identify immune fingerprints that reflect a person’s lifetime exposome starting with prenatal disease prediction (61). The maternal immune activation process during pregnancy, as described in the next section, further explains etiopathogenesis that can impair the MPF triad. The DoHaD paradigm applied to immunological mechanisms can help predict prenatal risks for life-course neurologic sequelae (62, 63).

### Maternal-fetal infectious/inflammatory responses

Communicable diseases can result in maternal and fetal inflammatory responses in the placenta (64). Placental inflammatory pathologies expressed throughout pregnancy are represented by chronic villitis of unknown etiology, chronic deciduitis-chorionitis, eosinophilic T-cell vasculitis, and chronic histiocytic intervillositis. Acute or subacute clinical or histological chorioamnionitis/funisitis contributes to peripartum preterm and full-term delivery. This spectrum of time-dependent pathogen-related diseases involves two complex immunological disease pathways that impact the fetal immune system (65). The first involves mechanisms that contribute to injury from the upregulation of host immune responses associated with hypoxia-ischemia in the placenta, cord, and fetal blood–brain barrier, combined with the harmful excessive release of specific cytokine species. The second involves perturbation of HLA transcripts which increase risks for fetal semi-allogenic graft rejection with miscarriage or maternal-placental-fetal diseases. Neurologic sequelae are associated with both etiopathogenetic mechanisms (66).

Xenobiotic exposures adversely alter placental membranes and worsen morbidities after ascending vaginal or blood-borne infections affect the fetus through placental disease. Resolution therapy can reduce inflammatory effects but requires pregnancy protocols to assess treatment efficacy versus risk that reduces the incidence of preterm delivery with less fetal brain injury. Adverse effects from climate change disproportionately affect the most vulnerable women and children with health disparities who are exposed to infections (67). A growing list of congenital infections associated with childhood neurologic sequelae requires greater attention from public health prevention and intervention programs (68).

### Mental health disorders and substance exposure

Recreational and prescribed controlled substances can be proxies of lifestyle choices and mental health disorders that contribute to TSI. Excessive corticosteroid and adrenergic hormone surges result from dysfunction of the hypothalamic–pituitary–adrenal axis during stresses from maternal mental illnesses. Harmful effects from these substances exacerbate these effects. While cannabidiol is currently a popular choice with recreational and medicinal benefits (69), neurotoxic effects also result, particularly when combined with substance exposures such as alcohol, tobacco, amphetamines, and opiates. Prescribed CNS-active psychoactive and antiepileptic medications also act as xenobiotics with similar undesirable neuronal effects (70, 71).

### Harmful effects from treatment interventions

Systemic disease treatments during pregnancy inadvertently contribute to fetal brain injury. Antibiotic administration to treat maternal genitourinary infections, for example, potentially alters fetal gut microbiota and impairs fetal neuronal cell development through exaggerated inflammatory disease pathways across the developing fetal neurovascular unit. Treatments may also disrupt placental inflammatory pathways that further contribute to fetal brain maldevelopment. Prenatal exposures later predispose the preterm neonate to systemic diseases such as necrotizing enterocolitis and sepsis (72). Postnatal brain injuries result through inflammatory, oxidative stress, and asphyxial neonatal disease pathways.

### Xenobiotic exposures with climate change

Uncontrolled use of organophosphate pesticides representing common agricultural residues contributes to neurologic diseases (73). Greater adverse effects result from global climate changes that reduce human health (67, 74). Altered gene expression with upregulation of pro-inflammatory cytokines, for example, contributes to non-infectious heightened inflammatory responses from xenobiotic-induced climate changes that impair developing fetal brain regions. Worsening exposures continue between pregnancies during childhood into adulthood. Public health interventions that address climate change are required to improve health care for women, children, and adults.

### Social determinants of health

Vulnerable maternal populations with healthcare disparities carry greater risks for neurologic sequelae, worsened with xenobiotic exposures. Poverty with food and home insecurity amplifies adverse disease effects, particularly for persons of color (75, 76). Public health programs need to effectively educate stakeholders regarding TSI risks within rural and urban communities. Monitoring levels of toxic exposure through governmental oversight need to be shared with women and their families in high-risk communities. This information disseminated through public health programs strengthens shared decisions with healthcare providers to improve person-specific health and quality of life.

**Figure 3:**
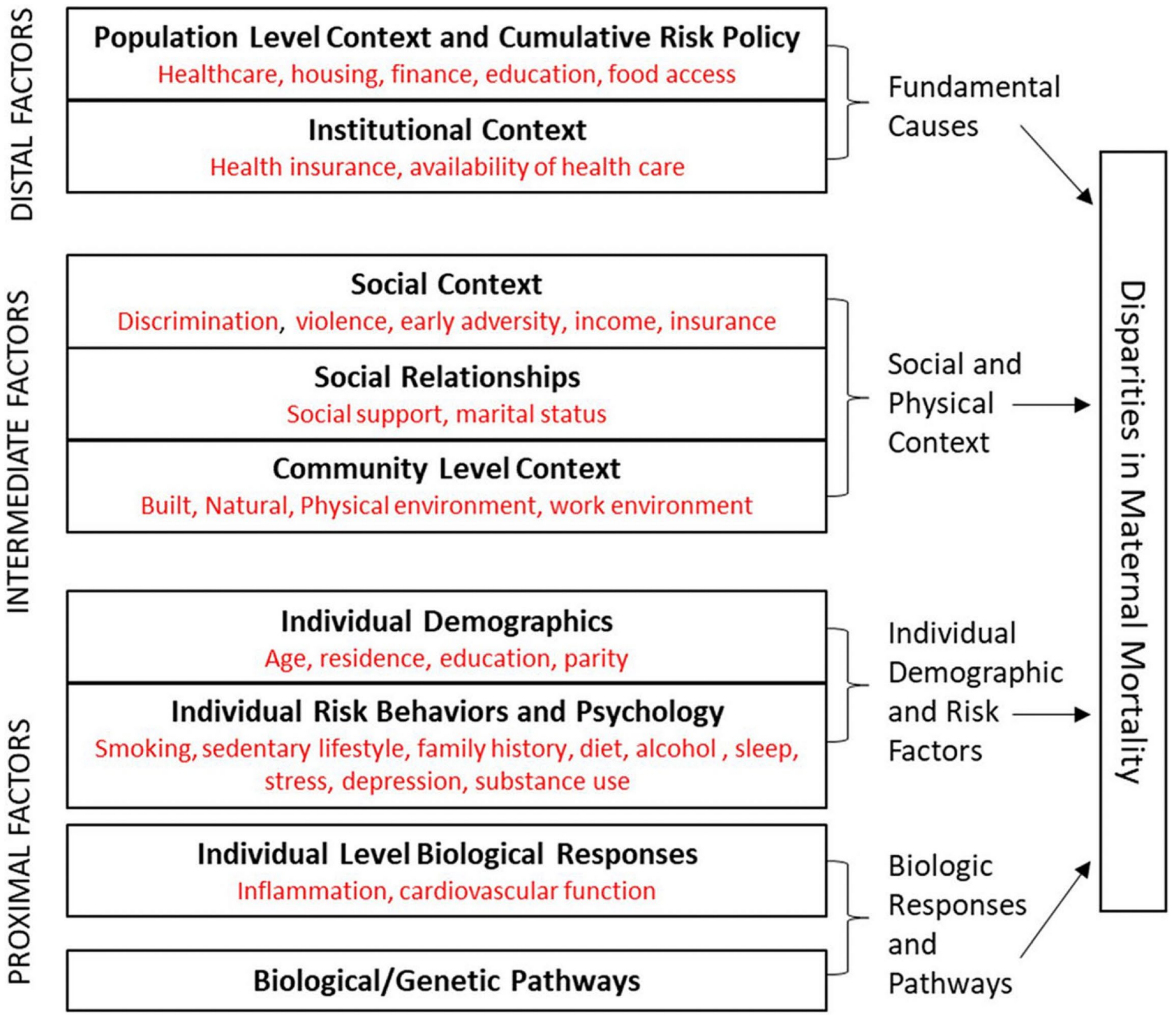
A conceptual model for disparities in maternal mortality suggest a complex interaction among proximal, intermediate, and distal factors that more globally explain healthcare disparities. Biologic responses and disease pathways as well as individual demographic and risk factors comprise the proximal factor category. Social and physical context comprise the intermediate factor category. Fundamental causes of disease are represented by the distal factor category. Reprinted with permission Harville et al. (43) licensed under CC by 4.0.

A recent computational analysis to assess the public health exposome evaluated factors that correlated specifically with pregnancy-related mortality. This study provides strong evidence that maternal death exemplifies one among a broad spectrum of women’s health behaviors, social determinants, and environmental exposures that influence adverse outcomes. The effects of these factors can be assigned to proximal, intermediate, and distal levels of risk that also pertain to maternal morbidities (43) (Figure 3).

### The concept of matrescence

**Figure 4:**
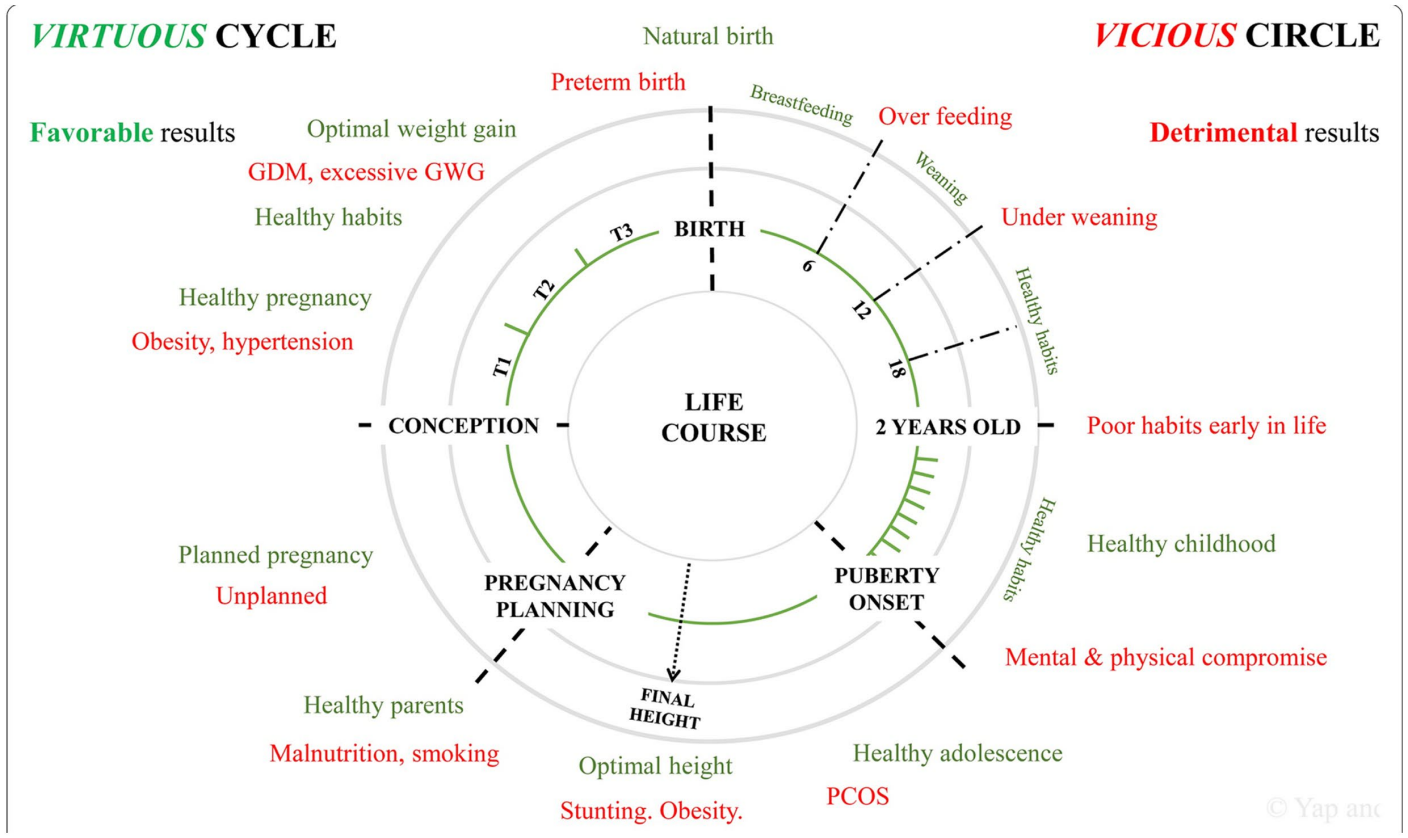
Favorable (“virtuous”-green color) health practices reduce the adverse effects from unfavorable (“vicious”-red color) health practices across the lifespan beginning with reproductive followed by pregnancy health. GDM, gestational diabetes mellitus; GWG, gestational weight gain; PCOS, polycystic ovarian syndrome; T1, first trimester; T2, second trimester; T3, third trimester. Reprinted with permission Yap et al. (78) licensed under CC by 4.0.

The growing field of matrescence emphasizes a woman’s adaptation during one or multiple pregnancies (77). Immediate and long-term positive or negative stressor responses influence a woman’s brain health during each pregnancy as well as beyond her reproductive years. Resilience or vulnerability to TSI will consequently affect the woman and her family’s abilities to care for their children, particularly for those challenged with neurologic disorders. A recent model-of-care framework presents an integrative life-course approach to health maintenance for women inclusive of preconception, pregnancy, and postpartum phases of healthcare. A social-ecological model identifies individual, interpersonal, institutional, community, and policy as major targets that promote health interventions for the woman-child dyad, supported by her partner and family (78)(Figure 4). Addressing reductions from xenobiotic exposures needs to be part of this effort.

## The placental exposome

The placental exposome contributes to fetal brain disease which includes TSI effects on the MPF triad (79). FNN trainees require familiarity with perinatal pathology applicable to developmental neuropathological effects as expressed by neurologic phenotypes during the first 1,000 days. Knowledge of time-dependent placental disease pathways better anticipates expressions of neurological disorders that later appear throughout childhood and into adulthood.

TSI can disrupt placental functions required for energy-substrate delivery, waste removal, and growth factor production and transport (80). Impaired progenitor neuronal populations result from placental disease pathways during the first half of pregnancy expressed as dysgenesis. Placental diseases during the latter half of pregnancy are more likely to contribute to destructive fetal brain lesions. Maladaptive placental epigenetic G × E interactions contribute to fetal brain anomalies or destructive lesions, dependent on trimester-specific TSI effects on the MPF triad.

Three disease pathways will be discussed that involve hypoxia-ischemic, oxidative stress, and inflammatory adverse effects by the placental exposome that can injure the fetal brain. Brain-placental axis communication combines biological susceptibility with pregnancy-related diseases and is worsened by xenobiotic exposures (81). Histopathological identification of these three placental lesion categories provides postnatal opportunities to consider etiopathogenesis involving TSI between the brain and placenta that correlates with neurologic sequelae (82). Future structural and functional placental-brain imaging modalities (83, 84) using fMRI combined with exomic/high through-put genetic testing will offer prenatal opportunities for diagnosis and interventions (83).

**Figure 5:**
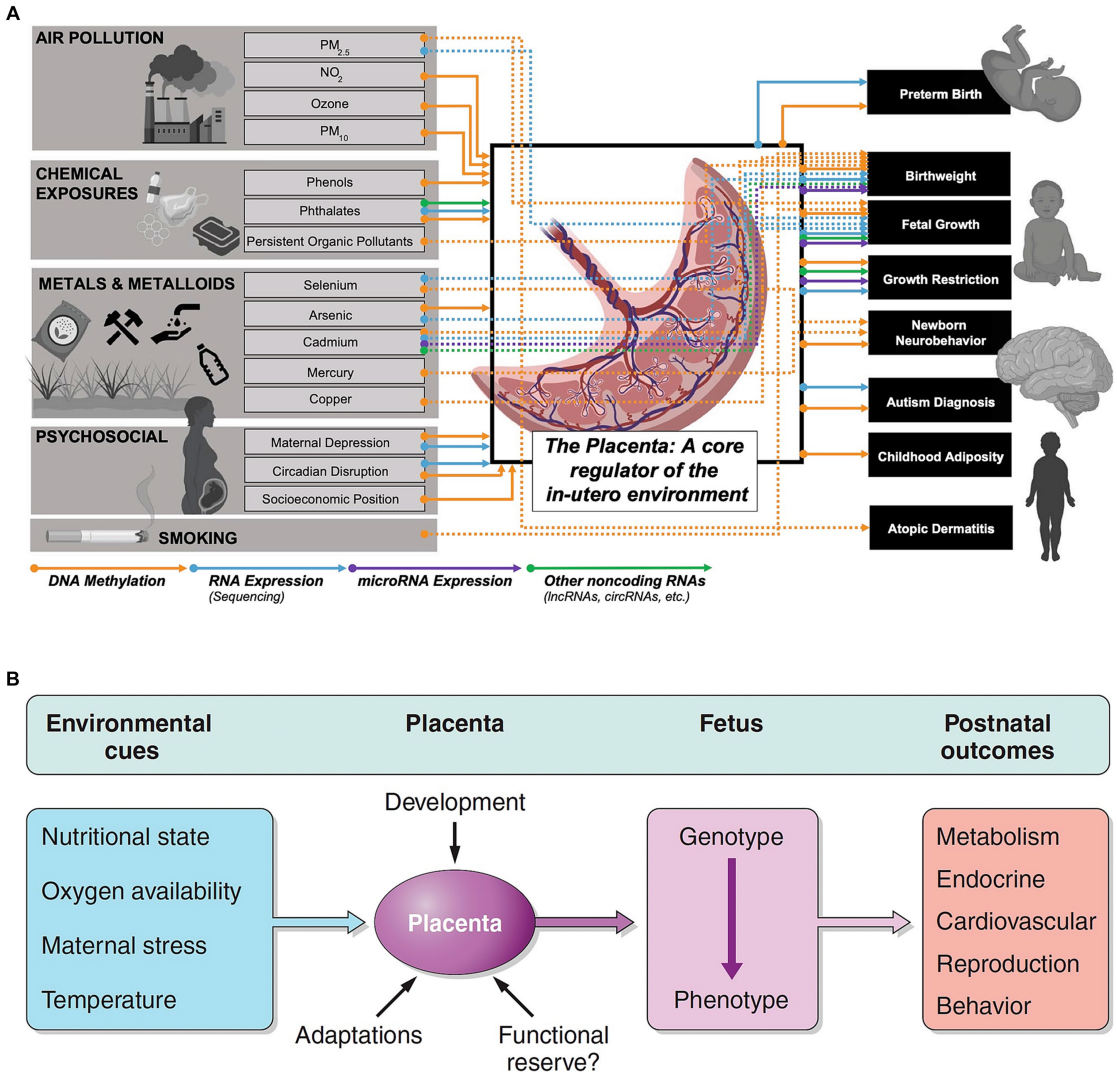
**(A)** Associations and connections between placental exposures with birth/early childhood health outcomes can be measured through placental epigenomic technologies, such as methylation and mRNA expression. Adverse effects from exogenous sources such as particulate matter (PM) and endogenous factors such as maternal stress responses represent a more comprehensive list of biological-chemical interactions that comprise the totality of toxic stressor interplay regulated by the placenta. Reprinted with permission Laephn et al. (85) licensed under CC by 4.0. **(B)** Diagrammatic illustration showing how the placenta may modulate and transduce environmental cues that lead to developmental programming of the fetus with effects later in life. The functional capacity of the placenta will depend on trimester-specific development, and the ability to adapt to stressors with existing reserves. Reprinted with permission Burton et al. (86) licensed under CC by 4.0. Vulnerability or resilience to stressors results in different life course expressions of abnormal neurologic outcomes.

The placental epigenome also helps identify molecular biomarkers that link prenatal exposures with fetal or childhood health outcomes (85) (Figure 5A). These same complex placental mechanisms affect the MPF triad and contribute to the developmental programming of chronic disease later expressed through reproductive senescence (86) (Figure 5B). Placental dysfunction may be active without detectable structural placental lesions through mechanisms involving xenobiotic transporters and metabolizing enzymes affecting biological-chemical interactions (87).

The remainder of this section will describe maternal immune activation, ischemic placental syndrome, and fetal inflammatory response that exemplify three major placental pathological processes influenced by TSI.

## Maternal immune activation

Maternal immune activation (MIA) is a placental disease process with adverse effects following placental implantation of the blastocyst between 2 and 8 days following fertilization. Suboptimal attachment of the blastocyst at the maternal-fetal uterine interface results in immune intolerance from TSI between the embryo as the semi-allogenic graft to the mother as the host (88). This inflammatory disease pathway impairs precursor trophoblastic cell development. Pregnancy termination with miscarriage occurs or the surviving embryo suffers from multi-system impairments that threaten fetal health. These earliest adverse effects can impair embryonic transient brain structures. Abnormal early microglial precursors originate within the yolk sac and contribute to cellular pathways resulting in brain maldevelopment (89). Neuronal dysgenesis continues after 17–19 days of gestation following the formation of the neural tube. Worsening effects to maturing fetal brain structures from MIA will continue as the functional hemochorial placenta replaces the yolk sac after gestational weeks 10–12. Later placental dysfunction through term gestation contributes to destructive brain lesions resulting from placental pathologies such as vasculopathies and villous dysmaturity.

Effects of MIA during the first half of pregnancy impair precursor neuronal and glial cell populations across the proliferation, differentiation, and migration stages, involving important transient brain structures such as the marginal and ventricular zones as well as the ganglionic eminence (90). Dysgenesis from MIA continues to be expressed during the second half of pregnancy, affecting more mature fetal brain structures such as the subplate zone and cortical plate, disrupting the formation of the six-layered cerebral cortex.

MIA-associated alterations in fetal brain development are later expressed as autism spectrum disorder, cerebral palsy, epilepsy, and behavioral/cognitive disorders during childhood (88). Conventional brain MRI magnification often detects only nonspecific markers of dysgenesis, represented by gliosis and atrophy. More precise identification of anomalous cortical development requires the next generation of fetal neuroimaging techniques that combine structural with functional assessments using brain fMRI and volumetric imaging (91, 92).

Reprogramming of the fetal brain and immune systems through inflammatory, oxidative, and hypoxic mechanisms associated with MIA continues throughout pregnancy, influenced by TSI (62). Epigenetic effects on fetal brain development involve interactions among microglia, peripheral immune system development, and colonization of gut microbiota. Inflammatory and non-inflammatory MPF factors induce the release of pathogen associated molecular patterns (PAMPs) and damage associated molecular patterns (DAMPs). These patterns activate toll-like receptors on maternal peripheral innate immune cells and placental cells that alter gestational-age-specific cytokine species. Passive transport and active placental production of these abnormal immune mediators cross the placenta to adversely interact with the fetal brain through metabolic, neuroendocrine, and stress signaling pathways. Maternal inflammatory effects resulting from MIA induce epigenetic memory within fetal microglia and immune cells throughout pregnancy. Fetal brain lesions are worsened by priming effects following endogenous maternal stressors (93) or xenobiotic exposures (94). Double-hit phenomena after the initial prenatal MIA are later associated with postnatal disease experiences. Childhood developmental disorders and epilepsies and neurodegenerative sequelae following reproductive senescence are long-term expressions of MIA effects during pregnancy.

Dynamic peripheral-central immune crosstalk is particularly enhanced during critical periods of neuroplasticity, beginning before 2 years of age and later followed during adolescence and reproductive senescence. Peripheral inflammatory signals continue to be triggered by environmental immune modifying TSI during childhood, with adverse activation of brain immune cells. Aberrant immune programming, genetic risk, sexual dimorphism, and multiple life-course hits cumulatively contribute to chronic inflammatory alterations within more mature and aging brain and peripheral systems.

## Ischemic placental syndrome

Ischemic placental syndrome (IPS) is the second placental disease category in relation to TSI. Precursor trophoblastic cellular maldevelopment during the first trimester (58, 59) results in defective angiogenesis comprised of shallow spiral artery remodeling. Limited vascular penetration into the placental bed following 8–12 weeks GA reduces blood flow to maternal and fetal surfaces, impairing decidual tissue or chorionic villi. Thrombotic lesions on the maternal surface or avascular villi with thrombi on the fetal side are representative malperfusion lesions. IPS may worsen as MPF triad diseases during pregnancy continue, such as those associated with gestational diabetes (56, 57, 95) or PIH (55). Increased risks for fetal brain injuries result, with or without coincident fetal growth restriction as a sonographic biomarker (96).

Umbilical cord lesions contribute to the reduction of oxygen, glucose, growth factor delivery, and waste removal from the harmful effects of IPS on the fetal brain. Two embryonic stalks connect the yolk sac to the embryo, comprised of the vitelline duct and vessels and the allantois and umbilical vessels (97). Marginal or velamentous umbilical cord insertion, as well as true knots and excessive coiling indices (98, 99), represent anomalous first-trimester cord maldevelopment. Narrowed vascular diameters within these cord lesions result in high vascular resistance that reduces placental blood flow. These cord anomalies accentuate the effects of xenobiotic exposures from TSI associated with placental diseases such as IPS.

## Fetal inflammatory response

Fetal inflammatory response (FIR) is the third placental disease mechanism affected by TSI. Acute and chronic forms of infectious or noninfectious FIR have been proposed as one of four categories of histopathological lesions as described by an international consensus of pathologists (100). FIR type I is a more acute immune response mainly driven by the activation of monocytes, macrophages, and neutrophils. This disease process occurs prior to and during parturition. Two disease pathways potentially contribute to fetal brain injuries from FIR type I: (1) inflammatory mediator-induced (i.e., cytokine-induced) asphyxia after vasoconstriction which reduces blood vessel caliber size and contractility responsivity within placenta/cord and fetal brain (i.e., blood–brain barrier) microvasculature; (2) direct inflammatory mediator injury to mitochondria within the neuronal cytosol and/or altered genetic expression within nuclei resulting in cell death or dysfunction (1). FIR type II alternatively represents a milder chronic inflammatory response involving perturbation of HLA transcripts with threatened fetal semi-allograft rejection during the first half of pregnancy. FIR type I may be influenced by xenobiotic effects later during pregnancy (101) with dysregulation of epithelial cell and macrophage immune responses as a mechanism that disrupts the cervicovaginal barrier (102).

FIR type II can be associated with chronic villitis associated with events during the first half of pregnancy (103).

## Neonatal neurocritical care and the neural exposome

Reproductive and pregnancy health adversities may present as fetal distress during parturition followed by neonatal brain disorders. Neonatal encephalopathy, seizures, stroke, and brain disorders of prematurity (EP) are complex phenotypes representing antepartum, peripartum, and neonatal timing of disease pathways represented by diverse etiologies (1).

Abruptio placenta, cord prolapse, or uterine rupture are three recognizable sentinel events most strongly associated with an increased risk for intrapartum neurologic injuries. Fetal distress with or without sentinel events has been defined using the interpretation of three categories of fetal heart rate (FHT) pattern abnormalities. However, current testing modalities cannot reliably distinguish antepartum from peripartum diseases (1). While FHT pattern abnormalities guide obstetrical decisions to avoid or reduce risks for brain injury, interpretations are unable to predict the onset or worsening of brain lesions (104–106). FHT pattern abnormalities often represent antepartum timing of diseases associated with fetal brain injury.

**Figure 6:**
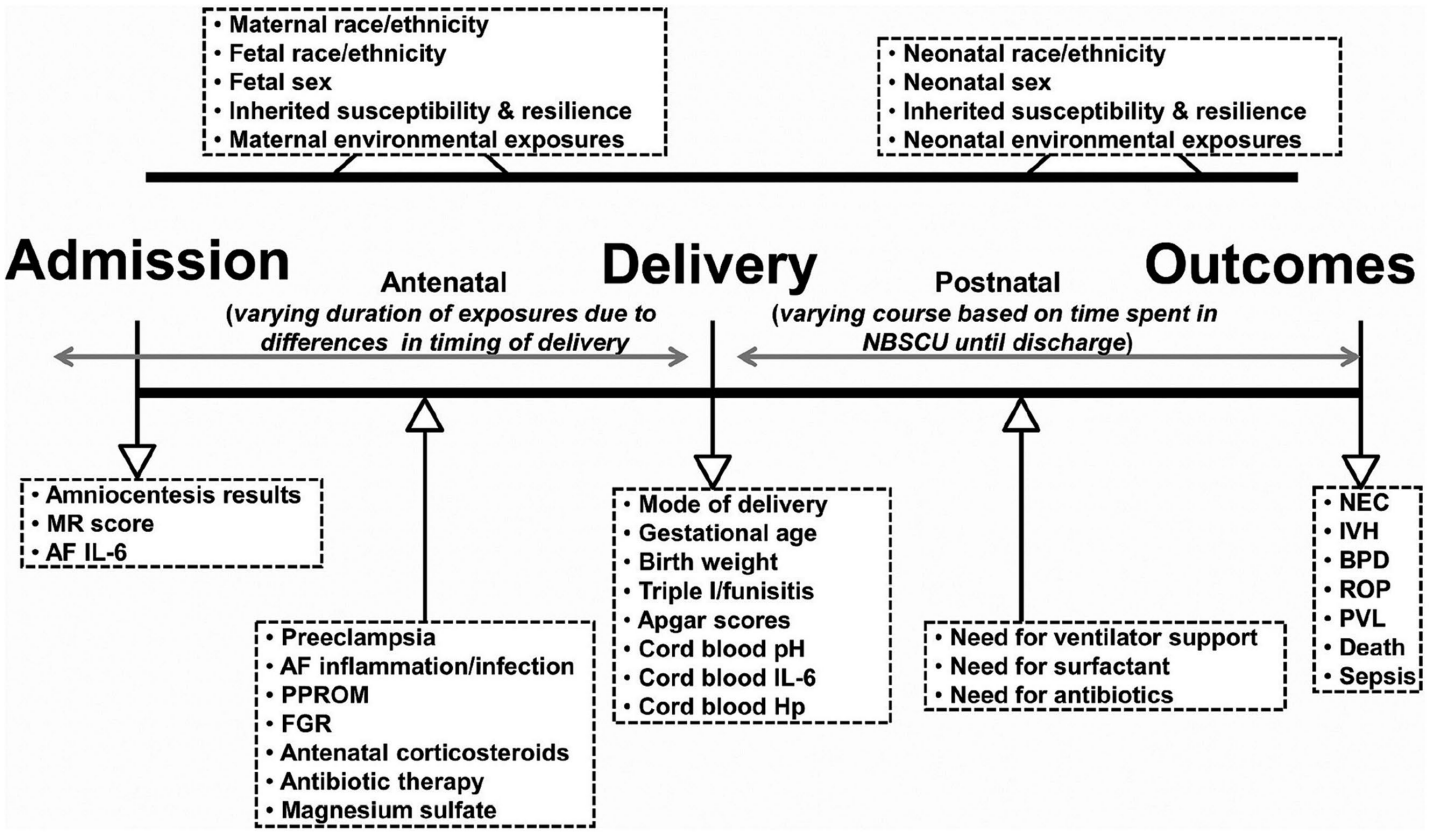
Schematic representation of fetal and neonatal exposome based on exposures and events that may occur in the antepartum, intrapartum, and postpartum periods resulting in prematurity. Clinical and laboratory variables informative of the exposome are listed inside the boxes. NBSCU, newborn special care unit; IVH, intraventricular hemorrhage; NEC, necrotizing enterocolitis; BPD, bronchopulmonary dysplasia; ROP, retinopathy of prematurity; PVL, periventricular leukomalacia; MR, mass restricted; AF, amniotic fluid; PPROM, preterm premature rupture of membranes; FGR, fetal growth restriction; Triple I, intra-amniotic infection/inflammation; Hp, haptoglobin. Reprinted with permission Nayeri et al. (110) licensed under CC by 4.0.

Neonatal encephalopathic presentations associated with depressed Apgars and metabolic acidosis on cord blood gas sampling are also important measures that guide intensivist care choices regarding resuscitative interventions. Therapeutic hypothermia (THT) and erythropoietin have been most extensively investigated. Future protocols will assess additional rescue agents (107, 108). Three clinical stages associated with encephalopathy and metabolic acidosis presently guide decisions to initiate THT. New protocols need to avoid past study limitations involving diagnostic biases regarding the identification of intrapartum hypoxia-ischemia. Reduced neuroprotective efficacy for many survivors who received these interventions was associated with nonmodifiable etiologies and/ or variable timing of the disease onset (109). Biomarkers are needed that more accurately represent a continuum of disease pathways associated with antepartum, peripartum, and neonatal TSI. Multisystemic diseases expressed by the MPF triad or neonate, particularly the preterm neonates, require appropriate neuroprotective interventions (110) (Figure 6). Present neonatal neurorescue options have limited efficacy, given the heterogeneity of neonatal populations based on different timing and etiologies (111).

Full-term and preterm newborns often express encephalopathy, seizures, and strokes based on trimester-specific MPF triad diseases. Each of these neonatal neurological syndromes includes consideration of secondary effects from TSI (1, 112). Serial neonatal diagnostic assessments until discharge offer greater insights regarding timing and etiologies, supported by neuroimaging and placental pathology findings.

## Neuroprotective rescue and clinical mimicry

Neurocritical care interventions help reduce the duration and severity of abnormal clinical repertoire which in turn may lessen the degree of sequelae. Emergency neonatal interventions are essential to treat cardiorespiratory complications from infectious or noninfectious etiologies. Medication and fluid administration help stabilize dysautonomia effects from systemic diseases that can worsen brain injury. Antibiotic or antiviral medications treat pathogen-specific etiologies expressed as sepsis and meningitis. Therapeutic hypothermia-erythropoietin rescue treatments may offer future treatment pathways to limit adverse effects for a percentage of newborns who were suspected of suffering acute intrapartum hypoxia-ischemia hours before delivery (107). Antiepileptic medications treat acute symptomatic seizures to avoid secondary dysautonomia effects associated with seizure-related brain injuries. Antepartum with or without peripartum contributions to fetal distress and neonatal brain disorders also need to be considered when choosing these interventions.

Clinical mimicry can be represented by each or any combination of the “great neonatal neurological syndromes,” encephalopathy, seizures, or stroke, for full-term and preterm neonates. Clinical mimicry from the secondary effects of TSI may be expressed by medication effects, neonatal abstinence syndrome, and multi-system diseases as discussed below.

## Medication effects suggesting clinical mimicry

Prenatal treatment with magnesium sulfate for PIH is the first of several examples of clinical mimicry of NE from medications. Antepartum brain lesions from remote adverse effects may contribute to acute encephalopathic presentations. Maternal administration of magnesium sulfate (113) into the fetal circulation contributes to varying grades of encephalopathy which correlate with serum magnesium concentrations. Reduced arousal and hypotonia mimic or accentuate clinical signs sometimes attributable to peripartum hypoxic–ischemic encephalopathy (HIE). These neonatal clinical signs may also reflect chronic MPF triad effects from PIH influenced by TSI. Placental lesions associated with IPS, as previously discussed, may be the result of biological-chemical interactions earlier during pregnancy. While some women who meet the criteria for preeclampsia are associated with fetal growth restriction, timing and etiologies associated with neonatal encephalopathy (NE) may remain inconclusive in the absence of this specific fetal presentation. These diagnostic challenges help explain why therapeutic hypothermia or erythropoietin administration does not effectively improve outcomes for many survivors (107, 114). While magnesium sulfate has been shown to have neuroprotective effects with lower rates of cerebral palsy in reported preterm newborn cohorts (115, 116), neurorescue for full-term neonates requires further investigation (117).

Perceived clinical signs of neonatal pain or distress by healthcare providers are assessed by subjective and nonspecific behavioral scales such as the Neonatal Infant Pain Scale (NIPS) and COMFORT behavioral scale (118). Neonatal nursing staff usually have the responsibility to assign scales to assess neonates subjected to uncomfortable medical-surgical procedures. Sedative-hypnotic drugs such as valium and fentanyl are chosen to treat perceived neonatal pain. These medications lessen encephalopathic signs without addressing timing or causal disease pathways expressed as NE.

Treatment with naloxone mitigates the severity and shortens the duration of clinical signs associated with neonatal abstinence syndrome. This treatment improves patient safety and outcomes by reducing adverse acute toxic effects on multiple organs. However, neuroprotective properties that target direct toxic brain effects following prenatal substance exposure have not been evaluated (119).

Substance use is often also a proxy for maternal mental health disorders. These women release excessive endogenous stressors based on hypothalamic–pituitary–adrenal dysregulation starting at conception. Harmful neurologic effects continue with excessive endogenous corticosteroid and noradrenergic substance exposures to sensitive embryonic and fetal brain structures. Placental disease pathways (i.e., MIA, IPS, and FIR) are also more likely to develop and contribute endogenous stressor effects through hypoxic, oxidative, and inflammatory disease pathways. Anomalous or destructive fetal brain lesion injuries can result dependent on the timing of stressors during pregnancy that impair fetal brain (120, 121).

Women confronted by health disparities experience greater TSI, worsened by co-existent neuropsychiatric disorders associated with substance use. Increased risks for abnormal maternal-child outcomes during the recent opioid and COVID pandemics have been experienced by women challenged with healthcare disparities. Knowledge gained from the HEALthy Brain and Child Development Study (HBCD) study in the United States will help improve health policy delivery and guide the development of interventions. One of the study’s objectives is to identify neurodevelopmental consequences from multiple adversities, including poverty, deprivation, and structural racism (122).

Neonatal seizures represent a third phenotypic presentation associated with medication effect representing NE mimicry. Dysautonomia results from the use of antiepileptic medications (AEDs), superimposed on encephalopathic signs from trimester-specific MPF triad diseases (1, 123). Algorithms to consider the discontinuation of AEDs have been presented (124). Any protocol requires person-specific applications to avoid seizure-related brain damage after premature discontinuation (125). Neonates with a greater seizure burden after HIE are more likely associated with MRI abnormalities (126). A minority of survivors with acute symptomatic neonatal seizures experience childhood epilepsies associated with cognitive-behavioral problems (127). However, increased seizure burden associated with more severe neurologic sequelae has been associated with prematurity (128) and specific etiologies such as central nervous system infections (129).

## System-specific neonatal diseases with mimicry

System-specific clinical scenarios highlight another example of NE mimicry based on prenatal origins of TSI. As the most common fetal malformation, children with congenital heart disease (CHD) represent trimester-specific G x E interactions affecting the neural exposome with TSI effects (130). Inherited syndromes and/or acquired diseases associated with CHD affect the MPF triad which in turn impair fetal and neonatal brain. Brain dysgenesis is often superimposed on destructive lesions when congenital heart disease is identified in the fetus, depending on fetal gestational maturity. These brain lesions are often correlated with placental disease pathways after pathological analysis is performed after birth. Both types of brain lesions contribute to neurological sequelae.

Different cardiopulmonary conditions adversely influence the neural exposome. Persistent pulmonary hypertension of the newborn (PPHN) includes acute disease pathways contributing to asphyxia resulting from vasoconstriction after meconium aspiration, often superimposed on peripartum hypoxia-ischemia. Chronic vascular remodeling and/or anomalous lung development after G x E interactions from TSI contribute to antepartum disease effects on the developing pulmonary system (131). Respiratory distress syndrome and lung anomalies such as congenital diaphragmatic hernia are other examples of neonatal cardiopulmonary conditions associated with brain injuries following TSI during pregnancy (132).

Neonatal sepsis and fetal inflammatory response syndrome represent multisystemic diseases that contribute to acute and chronic brain lesions. Preterm neonatal cohorts have comparatively higher percentages of inflammatory disease complications (110, 133) than full-term newborns. However, brain injuries at all gestational ages can result from inflammatory effects even without clinical chorioamnionitis or neonatal sepsis. An “omics” approach using diagnostic biomarkers of inflammatory pathways will more accurately detect asymptomatic MPF triads and neonates and offer pre-clinical therapies to improve outcomes (133).

## Developmental care influences the neural exposome

Altered state regulation from diseases, environmental conditions, and medications results in pain or distress perceived by providers and families. Physician, nursing, and therapist collaborations with families can lessen hostile NICU sensory environmental effects. NICU redesign better accommodates neonatal sensory sensitivities (134). Family engagement supported by the neonatal healthcare team helps mitigate TSI effects and better prepare the child for continuity of care following discharge (135).

Developmental care priorities should be introduced as early in the hospital course as deemed clinically safe and appropriate (136). Environmental adjustments to light, sound, touch, and sleep are major nonpharmacological developmental care interventions that are being prioritized for preterm and full-term neonatal care (136). All healthcare providers require skills to provide situation-specific interventions to support mothers and families. Skin-to-skin contact (i.e., kangaroo care) as well as massage and music therapies exemplify healthcare practices that have positively influenced brain health (137, 138).

Developmental care must be integrated into discharge plans for neonates born in low-middle-income countries (LMICs) and high-income countries (HICs) (135). Optimal nurturing practices benefit healthy infants as well as survivors of medical complications, particularly before 2 years of age. Neuropalliative care for the most medically fragile children should also include these interventions. Family involvement using these interventions enhances positive effects on neurodevelopment long after discharge. Knowledge of the exposome by family practice, obstetrical, and pediatric healthcare providers strengthens primary care wellness programs for mothers, families, and children that can reduce adverse experiences before and during pregnancy (139, 140). These practices help lower risks of sequelae starting during the initial critical/sensitive neuroplasticity age interval before 2 years of age (8).

## Neuropathology correlates of TSI

### First half of pregnancy

Variations in structural and functional brain development represent maturational effects on the neural exposome over the first 1,000 days. TSI adversely affects reproductive and pregnancy exposomes, contributing to anomalous or destructive fetal brain lesions through trimester-specific disease effects on the MPF triad (141). Brain maldevelopment initially results after disordered neurogenesis, differentiation, migration, apoptosis, axonal connections, and synaptogenesis, triggered directly or through epigenetic dysregulation. Examples of anomalies and disease processes include neural tube defects (dorsal induction), holoprosencephaly (ventral induction), microcephaly or megalencephaly (neuronal proliferation and apoptosis), lissencephaly, cobblestone malformation, or heterotopia (neuronal migration), and polymicrogyria or cortical dysplasia (cortical organization).

Sonographic screening during the first half of pregnancy may detect larger CNS malformations. Anomalies alternatively are initially detected or appear better defined during the latter half of pregnancy as well as after birth. Fetal neurology consultations with interdisciplinary input from neuroradiology, neurosurgery, and genetics offer more effective shared decisions among stakeholders when brain malformations are considered (142). Referrals to the FNN service optimize continuity of care from fetal to neonatal life for more effective interventions, prognostic predictions, and family counseling for future pregnancies. Significant neurodevelopmental and epileptic sequelae should be anticipated during the first 2 years of life.

Aberrant progenitor neuronal populations within transient brain regions during the first half of pregnancy result from G x E interactions that begin shortly after conception. TSI influences asphyxial, inflammatory, and oxidative disease pathways (142, 143), altering embryonic and early fetal brain structures within the first-trimester MPF triad. Dysgenesis may be expressed as focal, multifocal, or diffuse anomalous patterns. These lesions often are accompanied by excessive neuronal apoptosis with atrophy and reactive gliosis.

Large hemispheric, multifocal, or focal gray and/or white matter brain lesions may be visualized using current fetal sonographic and brain MRI technologies. Malformations of cortical development (MCD) comprise over 80% of discernable brain lesions initially detected by prenatal sonographic screening. Nonspecific anomalies such as ventriculomegaly and callosal dysgenesis often require fetal brain MRI studies to further delineate anatomical details, particularly during the late second or third trimesters. It may alternatively be preferable to obtain postnatal imaging given the woman’s stress and discomfort later during pregnancy (144).

Lissencephaly, schizencephaly, and holoprosencephaly are examples of a detailed classification scheme of MCD. Genetic evaluations further characterize G × E interactions and better define phenotype–genotype profiles. Whole exome sequencing and targeted high throughput genomic sequencing may identify specific genetic disorders associated with these defects. Commercially available genetic panels may suggest classical Mendelian or complex patterns of inheritance. Tubulinopathies exemplify one of the multiple complex neuronal disease pathways that represent genetic defects involving cell cycle abnormalities within the cytoskeleton of different neuroprogenitor populations (145, 146). Interdisciplinary efforts are now underway to establish prenatal diagnoses of tubulinopathies (147).

Post-translational modifications (PTMs) are comprised of a complicated sequence of spatially and temporally regulated processes required for healthy neurodevelopment (148). One process involves the regulation of the cytoskeleton and cytoskeleton-associating proteins, responsible for the stability, reorganization, and binding of microtubules and actin filaments. Dysregulations regarding this class of PTMs alter brain volume, dendritic growth, and composition with structural-behavioral defects. A second process involves gene regulation, ranging from chromatin modulation to protein turnover and degradation. Critical gene expression during neurodevelopment ensures correctly matured cells. Dysregulation of PTMs for these pathways also contributes to morphological and behavioral phenotypes. A third process of PTMs involves cell signaling and signal transduction which are vital for cell migration and axonal guidance. Complex post-mitotic processes including mosaicism, imprinting, somatic variation, and epigenetic alterations further increase the number of diverse phenotypes.

Ventricular and marginal zones, ganglionic eminence (149), and the subplate zone (150) represent transient brain structures during the first half of pregnancy that are vulnerable to the teratogenic effects of TSI. Impaired progenitor neuronal/glial populations (151) alter proliferation, migration, differentiation, axonal arborization, myelination, and synaptogenesis with resultant dysgenesis within the maturing cortical plate and hindbrain. Three representative subpopulations of developing cortical neurons have been chosen to highlight impaired neuronal maturation affected by TSI involving different cell types and their interconnections.

### Glial development

Glial neurons are essential for adaptive brain activities across developmental stages during childhood followed by aging throughout adulthood. While astrocytes and microglia are fundamentally different in origin and function than glia, all cell types collectively represent similar developmental processes including gliogenesis, angiogenesis, axonal outgrowth, synaptogenesis, and synaptic pruning (152). Neuronal-vascular interactions within the developing neurovascular unit (i.e., blood–brain/CSF barriers) constitute one critical neurodevelopmental site where neurogenesis and angiogenesis interact (153). Given the important early influences of these processes, dysfunctional macroglia or microglia during the first half of pregnancy potentially contribute to significant brain disorders across the lifespan. The FNN trainee’s understanding of normal or abnormal developmental expressions of these neuronal subtypes reinforces an appreciation of life-course brain health or disease expression, applying the concept of developmental origins.

Macroglia (e.g., astrocytes and oligodendroglia) have essential roles for the developing brain through the ontogeny of neural communication required to maintain brain health. Elements of macroglia cell identity and function and their time of emergence across species emphasize the diversity of influences that begin during the first half of pregnancy, preserved over evolutionary time across species (154). Cortical radial glial cells, for example, are primary neural stem cells that transform into cortical glutaminergic projection pyramidal neurons, glial cells (oligodendrocytes and astrocytes), and olfactory bulb GABAergic interneurons (155). These cellular transformations occur within marginal and ventricular zones of the embryonic cortical plate beginning at gestational week 8.

Altered macroglia development by abnormal epigenetic effects from TSI can later occur despite healthy parental or conceptus genetic endowment (156). Immunohistochemical analyses have identified specific neuronal transcription factors within embryonic neuroepithelial (pallial) stem cells which contribute to different disease pathways. Through a series of mitotic transformations, altered expressions of epidermal growth factor receptor (EGFR), achaete-scute homolog 1 (ASCL1), and two types of oligodendrocyte transcription factors (OLIG2 and OLIG1) occur through gestational week 18.

Progression and transformation of these cell types form important interconnections over the first half of pregnancy with developmental consequences throughout the first 1,000 days. Specific astrocyte subtypes stress morphological and molecular features required to preserve brain health. Different brain disorders are expressed across the lifespan as developmental disorders and epilepsies, representing prenatal macroglia maldevelopment (157).

### Microglial development

Microglial precursors exemplify another important neuronal population. This cell type is required for innate immune memory throughout the lifespan with contributions to brain health or disease risk (158–160). Myeloid precursor cells originate within the yolk sac (89). Cellular migration is followed by neural plate formation after 17–19 days. Microglial precursors develop prior to the functional placenta, replacing the yolk sac to provide nutrient delivery, waste removal, and growth factor production after gestational weeks 8–12. A subpopulation of these embryonic microglia reacts to TSI with impairment of neural progenitors in a sexually dimorphic manner (161). The inflammatory responses mediated by altered microglia play important roles in ischemic brain injuries occurring later during pregnancy through different etiopathogenetic pathways. Knowledge of the activation, polarization, depletion, and repopulation of microglia after ischemic brain injuries will suggest fetal treatment strategies during the second half of pregnancy through the modulation of microglial function (162).

### Interneuron development

Interneuronopathies exemplify a third important early neuronal cell type disruption from TSI with lifelong neurologic consequences. Prenatal disease pathways represented by this progenitor neuronal population (163) contribute to excitatory-inhibitory (E/I) imbalance involving specific susceptible subtypes (164). Lifelong abnormal clinical consequences are significant given this cell population comprises at least 30% of all neuronal cell types distributed throughout the neuroaxis. Specific subgroups of interneurons such as GABA-ergic interneurons (165) contribute to diverse childhood neurologic diseases including autistic spectrum disorders, attention-deficit/hyperactivity disorder, mood disorders, and epilepsy. Early life E/I imbalance also contributes to cognitive, epileptic, and neuropsychiatric disorders presenting during later childhood into adult life. Accurate biosignatures of developing interneurons altered by TSI during prenatal life will help promote the development of neurotherapeutic interventions such as interneuron transplantations (166, 167).

Xenobiotics from air, water, and soil exposures (69) alter prenatal brain structure and function involving these different neuronal populations. Climate changes further exaggerate adverse genetic and epigenetic teratogenic effects (74). Dietary exposures from lead, cadmium, and mercury, for example, reduce specific central nervous system functions with bioaccumulation in bone and adipose tissue of the MPF triad with secondary effects on developing fetal neuronal subtypes. Fetal renal/hepatic functions are also consequently impaired, further decreasing critical functions of vital nutrients such as calcium, iron, and zinc needed by developing neurons. Primary teratogenic lesions during the first half of pregnancy are later worsened by TSI during the second half of pregnancy with continued exposures. Postnatal exposures continue to further impair neuronal circuitries.

### The second half of pregnancy

Neuropathological correlates during the second half of pregnancy result in destructive lesions affecting more mature fetal brain structures. Markers of hypoxic–ischemic, inflammatory, hemorrhagic, and thrombotic-embolic disease processes are documented by neuroimaging or postmortem neuropathological analyses. Abnormalities representing acute, subacute, and chronic neuropathological correlates (168) may be identified after serial neonatal neurologic assessments. Specific image interpretations using diffusion-weighted, T1, and T2 brain MRI sequences depict abnormalities that represent variable timing of neuropathologic processes representing acute cell death, pre-Wallerian degeneration, or liquefaction necrosis. These changes require hours to days of evolution to appear. Gliosis, atrophy, and cystic degeneration alternatively suggest longer time courses over weeks to months.

Spatially and temporally restricted neuronal development involves time-sensitive programmed cell death (PCD) signaling events that are required for normal structural and functional brain development (169). Apoptosis, necroptosis, pyroptosis, ferroptosis, and cell death associated with autophagy represent multiple combinations of abnormal PCD signaling cascades associated with etiopathogenesis during the first 1,000 days. Different forms of cell death can be activated in response to TSI represented by destructive lesions during more mature gestational ages. Activation of PCD pathways results in unwanted loss of neuronal cells while inactivation of PCD contributes to unwanted overgrowth, increasing vulnerabilities within specific brain regions. Identification of the mechanisms involved in the inhibition or induction of PCD after TSI will help identify critical disease pathways that are amenable to therapeutic interventions.

Later extensive destructive lesions after asphyxia, intravascular thrombosis, and hemorrhage obscure earlier anomalies. Diseases that exemplify such complex brain lesions include more prevalent maternal thrombophilias (170) to less common fetal stroke syndromes (171) and rare genetic defects such as CoL4A1–2 genetic mutations (172).

TSI will affect vulnerable fetal brain regions based on the predominant disease pathways that are active during that stage of fetal brain development. Gray and white matter lesions vary based on gestational age maturity as well as neuroanatomic site relevant to the onset and evolution of disease and etiopathogenesis.

### Peripartum and neonatal time periods

Peripartum and neonatal brain lesions represent the onset or worsening of a disease process. Acute and chronic disease pathways during parturition and neonatal life may contribute to the neuropathologic correlates. These consequences are based on similar but diverse etiopathogenetic mechanisms during prenatal life. Serial neurodiagnostic assessments through intensive care discharge combine examination, body-fluid, neurophysiological, neuroimaging, and placental pathology findings to approximate the most accurate diagnostic conclusions. A wider choice of future neurotherapeutic interventions will be needed, given the variable timing and etiology of neonatal brain disorders (114).

### Postnatal ages up to 2 years and beyond

**Figure 7:**
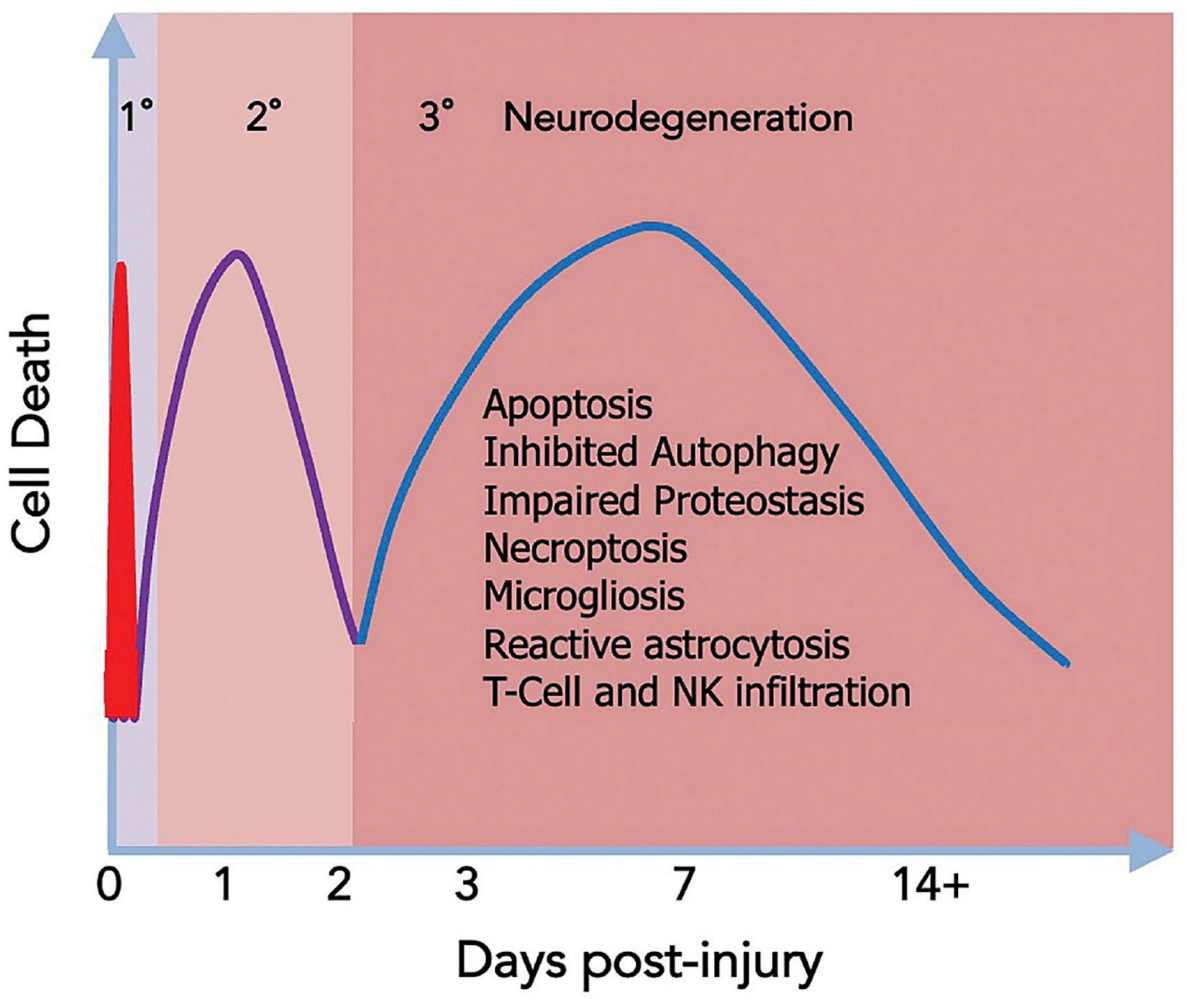
This figure summarizes the stages of neurodegeneration represented by neonatal hypoxia-ischemia. Potential injury mechanisms include primary, secondary, and tertiary phases. Reprinted with permission Levison et al. (173) licensed under CC by 4.0. Given the potentially long interval between the onset of tertiary neurodegeneration with completion, there are opportunities for neurotherapeutic interventions both before and after birth.

While different mechanisms contribute to acute and subacute brain lesions, tertiary stages of recovery involve cellular and molecular changes that extend weeks to months after prenatal or postnatal brain injury. Following primary and secondary degenerative changes, tertiary neurodegeneration is a significant contributor to neurologic sequelae (Figure 7). Pharmacologic interventions that lessen the effects of apoptosis, necroptosis, autophagy, protein homeostasis, inflammation, microgliosis, and astrogliosis were reviewed based on a limited number of preclinical and clinical studies pertaining to asphyxia (173). Greater focus using knowledge of this tertiary phase of neurodegeneration provides opportunities to develop neurotherapeutic protocols. These interventions include prenatal applications for the MPF triad as well as for the child up to 2 years of age.

## The neural exposome throughout childhood

Most children present with neurologic disorders despite healthy prenatal and neonatal histories during the first 1,000 days. Pediatric wellness surveillance may detect younger children with more obvious developmental-behavioral and paroxysmal disorders. Diagnostic accuracy must be maintained beyond 2 years of age using examination and diagnostic testing specific to the child’s age at clinical presentation. Communicable and noncommunicable clinical scenerios (14) often initiate investigations based on the predominate neurologic signs and symptoms. Infections, accidents, and systemic illnesses may have initiated acute injury as well as alternatively worsened pre-existing brain lesions (1, 174). Coordination between primary care practitioners and pediatric subspecialists requires ongoing vigilance to select the appropriate evaluations throughout childhood.

Adverse effects of the neural exposome expressed as neurologic disorders represent a wide range of endogenous and exogenous stressors experienced earlier by the pregnant woman and the younger child. The European-based Human Early Life Exposome (HELIX) study is a multi-institutional cohort designed to associate individual exposomic risks consisting of >100 chemical, outdoor, social, and lifestyle exposures assessed during pregnancy. Multi-omic profiles (methylome, transcriptome, proteins, and metabolites) of children between 6 and 11 years of age (175) were compared with these prenatal risks. This study protocol encompasses effects that can be assessed during childhood using psychometric measures.

Maladaptive developmental neuroplasticity may have altered the neural exposome from TSI during the first 1,000 days. Dysregulation of synapses and neuronal networks may be expressed by delays measured using less reliable developmental testing screening methods. Motor, language, cognitive and/or social-adaptive milestone delays often accompany primary sensory deficits in vision and hearing as expressions of neurodevelopmental disorders (NDD), notably autistic syndromes and intellectual disabilities. A battery of neuropsychometric and behavioral tests helps describe strengths and limitations with advancing ages. Preclinical animal models have advanced our understanding of typical and atypical neurobiological mechanisms. The neural basis of these neurodevelopmental disorders is expressed during the first critical-sensitive period over the first 1,000 days (176).

Mechanisms by which multiple factors diminish brain health are not fully known but most certainly involve synergy among multiple endogenous biological and chemical interactions affecting the reproductive and pregnancy exposomes. An NIH-supported program of 84 US cohorts entitled the Environmental Influences on Child Health Outcomes (ECHO) is conducting analyses through collaborative research projects to better understand the developmental origins of brain health or disease in relation to TSI, with proposed intervention strategies (177).

Oxidative stress, immune system dysregulation, altered neurotransmission, and endocrine hormone disruption alter gene expressions through genetic and epigenetic effects from adverse TSI effects beginning before conception and are worsened by ongoing childhood exposures. Current pharmacologic and nonpharmacologic interventions are more likely to mitigate rather than prevent poor outcomes later expressed as developmental, epileptic, or behavioral disorders.

*In utero* identification by mass spectroscopy and nuclear magnetic resonance will help reduce xenobiotic exposures such as from heavy metals and phthalates. More effective prevention or reduction of dysregulated neuronal metabolic pathways such as oxidative phosphorylation will be achieved. This particular prenatal neurotherapeutic strategy exemplifies time-sensitive approaches that will more effectively treat the fetus and lower the number of children who later express neurodevelopmental disorders (178).

Specific educational needs are required for each child according to individual strengths and limitations. Educational neuroscience principles (11–13) need to be applied as lesson plans are revised throughout the school years. Cognitive, language, behavioral, and motor domain challenges require individual educational plans for identified children with significant neurologic disorders (14). These goals also remain applicable for mainstreamed children who require behavioral and mental health interventions despite stronger cognitive skills.

TSI more likely contributes to neurologic sequelae for medically fragile children who experienced complications during pregnancy, neonatal, or early life. Healthcare disparities based on race, poverty, gender identity, and geographic isolation amplify negative effects on the neural exposome of these children (179). Brain health of children with complex multisystemic diseases is further worsened by negative family and social dynamics. Suboptimal parenting resulting from lifestyle choices or mental health disorders introduces adverse effects that contribute to more severely compromised outcomes, particularly for the most vulnerable children (9, 10).

## The neural exposome during adolescence

The developmental-aging continuum encompasses later critical periods of neuroplasticity during which significant alterations in brain structure and function occur. One transition builds upon the previous experience, based on consequential changes that occurred during the preceding developmental stage (180).

Healthy adolescent brain development results from adaptive mechanisms based on prior experiences throughout childhood that contribute to positive cognitive and social outcomes in preparing for adulthood. Disease states and risk-taking behaviors during adolescence alternatively contribute to maladaptive neuroplasticity effects that reduce adult performance and quality of life. Disorders of cognitive development, mood, and anxiety may occur even without co-existing medical conditions. Psychosocial challenges at home and school and within communities constitute sources of TSI during adolescence.

Medical, psychological, and social challenges during this critical period of maturation (181) are represented by a prospective longitudinal NIH-funded adolescent study in the United States. Targeted brain regions using quantitative brain MR imaging are being compared with neuropsychological scores to assess structural-functional correlates of cognition and behavior. Gray-white matter volumes and functional brain MRI activation patterns combined with cognitive-behavioral profiles assess the positive or negative effects of the neural exposome. Cognitive-social performances associated with intellectual-mental health disorders are specific outcome measures (182). The identification of effective interventions will facilitate successful transitions into adulthood.

This adolescent-centered study has been more recently joined by an NIH-supported early childhood study entitled HEALthy Brain and Child Development (122). This more recent multi-institutional effort includes scientific objectives that are examining targeted factors associated with TSI, highlighted by opioid exposure during the COVID-19 pandemic, and exacerbated by the harmful effects of healthcare disparities. Maladaptive neuroplasticity effects are being assessed as expressed by abnormal child development. Medical, social, and educational interventions will be studied that exemplify these two studies and better identify factors to minimize complications during adolescence.

## The neural exposome during aging

The developmental origins of health and disease (DoHaD) theory correlate early life TSI with adult diseases, exemplified by obesity, cardiovascular, and neurodegenerative diseases (183). Defective developmental neuronal programming results in adult adverse effects of metabolic syndrome which impair the aging nervous system (184). Delayed pathophysiological and behavioral deficits are clinically expressed and accentuated following reproductive senescence.

Long latencies between exposures, later onset of diseases, difficulties identifying predominant early exposures, and multiple later life factor interactions make the application of the principles of DoHaD challenging, given current epidemiologic or longitudinal study design limitations. However, two longitudinal cohorts are discussed to reinforce improved outcome prediction by applying the DoHaD concept to clinical practice.

The Icelandic AGES project demonstrated increased risks for neurologic morbidities at old ages as predicted by a subtle measure of fetal growth restriction (i.e., reduced ponderal index). This prenatal biomarker of TSI affecting the MPF triad predicted lower neuropsychometric performances and smaller gray and white matter volumes on brain MRI studies for survivors during their eighth decade of life (185). Greater educational experiences and positive lifestyle choices represented preventive healthcare practices that alternatively predicted more positive brain health.

A more recent longitudinal study reported a cohort during early adulthood used a machine-learning approach to assess structural brain changes by using quantitative brain MRI image analyses between 25 and 33 years (186). Imaging measures were correlated with quantitative behavioral scores that measured anxiety and depression. Adversities spanning the prenatal period up to adulthood were associated with persistent widespread neural signatures in the brain that remained stable over an 8-year interval. Brain changes were region-, adversity-, and timing-specific, demonstrating neuroanatomically distinct trajectories of brain expansions and contractions related to specific forms of TSI. Widespread individual-level deviations from the normative model were predictive of mental illness, indicating that inter-individual differences were clinically meaningful. These findings support advocacy to offer interventions for younger adults to improve public mental health based on earlier life risks.

These two studies illustrate how FNN training strengthens career-long neurologic practice to benefit older persons. Familiarity by adult neurologists of early life effects on younger adults as well as aging nervous systems can better select diagnostic and therapeutic strategies for persons experiencing cerebrovascular, epileptic, neuroinflammatory, and neurodegenerative disorders.

Recent animal models have assessed the long-term effects of early-life exposures to specific xenobiotics such as heavy metals and pesticides. These studies support the link between exposure and neurodegenerative diseases during older ages (187). MIA during early pregnancy demonstrates one placental disease pathway previously discussed. MIA represents early-life inflammatory effects from TSI with later-life neurologic effects after repeated exposures into adulthood. IPS and FIR during pregnancy can further enhance vulnerabilities to these later-life neurologic disorders, as represented by a multi-hit hypothesis.

Neurodegenerative diseases such as Alzheimer’s disease and Parkinson’s disease are adult-onset neurologic diseases that often present after reproductive senescence. These diseases have been correlated with early-life disease pathways beginning before birth. Increased neuroinflammation, oxidative stress, and glial activation in adult populations may have begun during prenatal life from TSI affecting the MPF triad, further exacerbated across the lifespan. Multi-omics technologies will more accurately associate specific stressors across ages resulting in remote effects (86) to offer more effective interventions.

Convergence of risk factors impairs cell/molecular pathways during critical developmental time periods that combine susceptibility-based genetic profiles with acquired diseases. Biosignatures consisting of genetic and epigenetic profiles will more accurately identify TSI that predict child and adult brain disorders (188). Research must consider environmentally relevant toxic doses, dose–response curves, and relationships among molecular mechanisms to more effectively study the origins of neurologic diseases that adversely affect reproductive and pregnancy health.

## Neurotherapeutics that target the neural exposome

Comprehensive neurotherapeutic strategies will require preventive, rescue, and reparative interventions across the lifespan to address the timing of TSI that threatens brain health. New research needs to more effectively detect endogenous and exogenous stressor effects with intergenerational and transgenerational neurological consequences (188). Bio-signatures are required to monitor biological-chemical interactions that directly cause brain injury, or indirectly cause damage through multi-systemic diseases. Comprehensive biomarkers must encompass synthetic chemicals, dietary constituents, psychosocial stressors, and biological factors, with testing prior to and during each pregnancy. High-resolution mass spectrometry (HRMS) and metadata informatics are examples of novel TSI assessments, linking neural exposome effects to environmental neuroscience. These diagnostic advances must be relevant to women’s and children’s health. Applying a white male standard to research design is both unscientific and unethical, given the exclusion of over half the population who are biologically female (189). A Human Exposome Project on a scale comparable to the Human Genome Project has been suggested (188). Diversity and inclusion priorities will be required when designing research trials (190).

The application of nontargeted analyses might improve the characterization of maternal and pediatric exposomes. These tests must be inclusive of reproductive and pregnancy surveillance of health threats (191) that negatively influence the MPF triad and child during the first 1,000 days. Subsequent monitoring of TSI continues to promote interventional strategies that preserve brain health over the lifespan. This is exemplified by the adjustment of a person’s neuroimmune responses to TSI with advancing age. This concept will help develop neurotherapeutic choices (192) that prevent or mitigate disease pathways. Given that multiple xenobiotic exposures express complex transcriptomic and metabolomic bio-signatures based on diverse toxin-toxin interactions, non-targeted person-centered body fluid analyses (193) can more accurately adjust to a person’s health or disease immune status across the development-aging continuum.

## Exosome-related therapeutic applications

**Figure 8:**
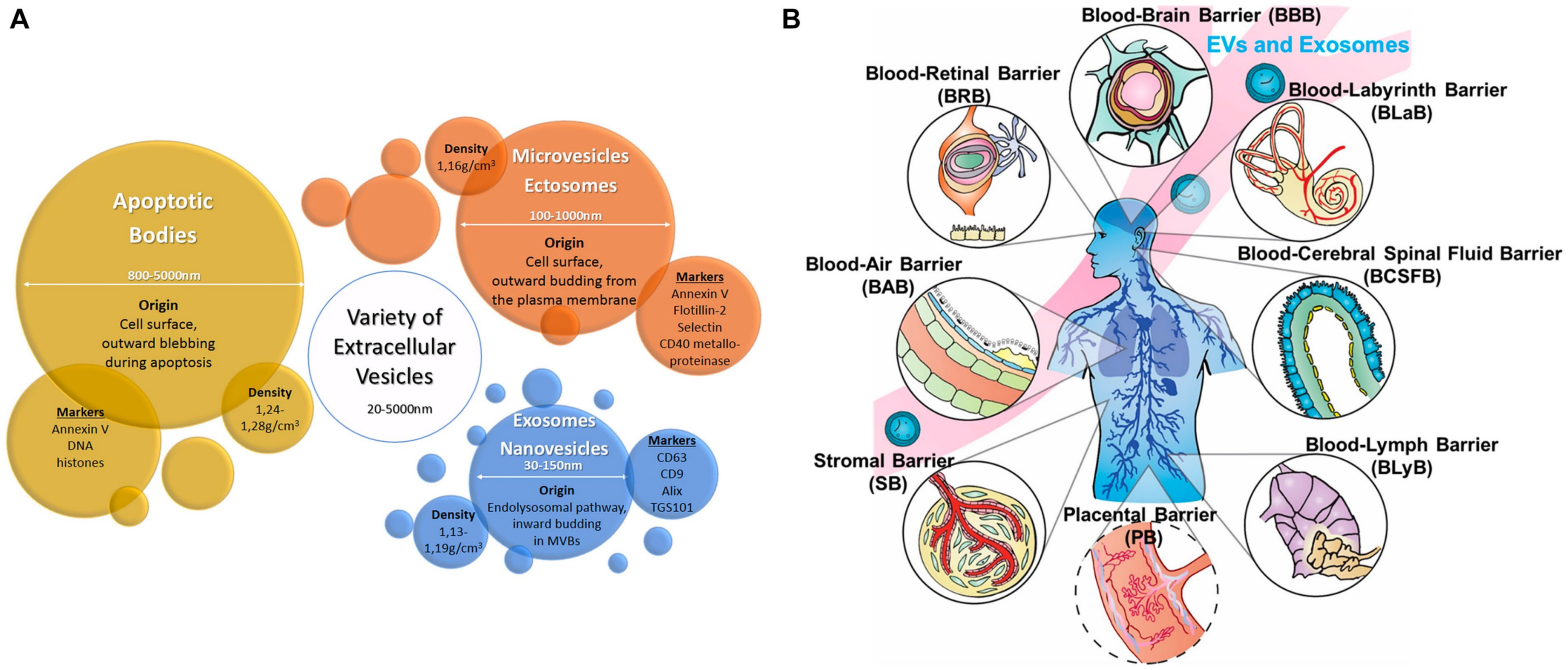
**(A)** Origin, size, density, and typical markers of major extra cellular vesicle subclasses. Reprinted with permission Czernek et al. (194) licensed under CC by 4.0. **(B)** Illustration of essential biological barriers penetrated by exosomes for cellular regulation and drug delivery. Reprinted with permission Elliott et al. (195) licensed under CC by 4.0.

Extracellular vehicles (EVs) are a heterogeneous group of cellular and membranous particles that could be harnessed for targeted drug delivery (Figure 8A). EVs originate from different cell compartments and represent essential physiological functions and therefore have distinctive properties in cellular information transfer and uptake. Exosomes easily cross biological barriers, overcoming obstacles that challenge drug delivery. Barriers include the blood–brain barrier, blood-cerebrospinal fluid barrier, blood-lymph barrier, blood-air barrier, stromal barrier, blood-labyrinth barrier, blood-retinal barrier, and placental barrier (195) (Figure 8B). These properties open exciting new possibilities for drug delivery platforms during pregnancy to maintain healthy maternal-placental-fetal communications (196).

Exosomes are the smallest unit of EVs released by the fetal brain as well as other organ systems, including placenta-cord tissues and their membranes. EVs are involved in angiogenesis, endothelial cell migration, embryo implantation, and transport of diverse molecular cargoes that influence physiological factors in healthy and pathological pregnancies. Alterations in exosome concentration, composition, and/or bioactivity include interactions with maternal cells through signaling pathways that may participate in pathological pregnancy states (194).

### Placental-derived exosomes

Placenta-derived exosomes (pEXO) for example are detectable in the maternal blood as early as gestational week 6 with increasing levels across pregnancy. Recent studies have demonstrated that pEXO play a key role in the establishment of maternal immune tolerance to maintain a healthy pregnancy (197). Altered molecular signatures of pEXO could be potential biomarkers for outcomes affecting the MPF triad from diverse conditions such as preeclampsia, fetal growth restriction, gestational diabetes mellitus, and preterm birth. Understanding the underlying disease mechanisms associated with specific pEXO abnormalities may help identify impaired embryonic and early fetal brain development that contribute to neurologic and mental health disorders.

pEXO biomarkers may be used for drug delivery to preserve healthy multipotential neuronal development through specific cellular pathways such as those mediated by TOR protein kinase (198). Following fetal neuroimaging identification of MCD correlated with specific prenatal whole exome sequencing profiles (199), neurotherapeutic use of rapamycin for example during early pregnancy might mitigate dysfunctional effects associated with tuberous sclerosis complex.

### Neuron-derived exosomes

Central nervous system intercellular communications are essential to preserve healthy brain maturation and homeostasis. Neural-derived exosomes are critical mediators of intercellular signal transduction (200).

Neural cells secret EVs including exosomes that influence multiple factors such as the transport of specific proteins, lipids, nucleic acids, and other bioactive molecules to recipient neurons that regulate functions under physiological and pathological conditions (201). Dependent on the CNS microenvironment relative to the physiologic or pathologic status of parental, placental, and fetal organ-specific cell communications, neural-derived exosomes mediate different effects, such as synaptic plasticity, nutritional metabolic support, nerve regeneration, inflammatory response, anti-stress effect, cellular waste disposal, and the propagation of toxic components. Neuron-derived exosomes play an important role in maintaining brain health during pregnancy and across childhood into adulthood. Harnessing the functions of neuro-derived exosomes will help monitor disease and assess therapeutic responses, including congenital infections (202), preeclampsia (203), fetal inflammatory responses with chorioamnionitis (204), and neonatal hypothermia after HIE (205), to neurodegenerative diseases at more advanced ages (206).

### Nutrition-based therapies

The importance of nutrition for development during the first 1,000 days encompasses macro-and micronutrients (207). More recent preclinical studies using rodent models of perinatal asphyxial brain injuries have (208) suggested the beneficial effects of dietary supplementation of polyphenols on neurological and behavioral parameters. Principal findings include (1) reduced region-specific loss of brain tissue, (2) decreased microglia activation and astrocyte activation, (3) reduced volume brain damage, (4) improved sensory-motor and cognitive function, and (5) reduced anxiety and depressive behavioral responses. These benefits were associated with positive neural responses by reducing oxidative stress and neuroinflammation in different brain regions including the cerebral cortex, striatum, corpus callosum, and hippocampus regions (CA 1 and CA 3). Treatment with polyphenols also inhibited caspase-3 activity. Continued beneficial effects from polyphenol use into adulthood have been based on permanent alterations in epigenetic regulation expressed by maturing phenotypes.

### Heavy metal reduction augments dietary benefits

An exposome paradigm through an integrated approach is investigating the impact of perinatal heavy metal exposures on child neurodevelopment in two cohorts carried out in Slovenia (PHIME cohort) and Greece (HERACLES cohort) (209). Links between *in-utero* and early-life exposures to heavy metals, metabolic pathway dysregulation, and neurodevelopmental disorders were suggested using urinary and plasma untargeted metabolomics analyses, followed by the combined application of *in silico* and biostatistical methods. Prenatal and postnatal exposures were indirectly related to healthcare adversities that can be modified by altering sociodemographic and anthropometric parameters using dietary supplementation.

Prediction and prevention of adverse TSI effects on reproductive and pregnancy health require targeted therapeutic interventions. However, progress toward understanding pregnancy disorders affecting the MPF triad such as preeclampsia lags behind advances regarding other aspects of human health (210). Investigations have been largely limited to hypothesis-generating approaches, constrained by attempts to treat a specific disease phenotype rather than consider etiopathogenesis involving TSI that adversely affects the neural exposome.

## The neural exposome promotes a brain capital strategy

Interdisciplinary studies of the neural exposome merge women’s and children’s healthcare goals for lifelong brain health (26). The promotion of a brain capital strategy has been advocated through the science of social-to-biological transitions (75). Present brain research, innovation, regulatory, and funding systems are currently siloed, creating obstacles to our understanding of the brain. Concepts involving the developmental-aging paradigm should interconnect neurologic and mental health needs given that these disciplines are inextricably integral (211).

Maternal-child health initiatives begin with reproductive and pregnancy health priorities with accumulated biological capital across the lifespan (212). The United Nations Agenda for Sustainable Development provided a shared blueprint for global peace and prosperity, first proposed during the early 1990s with yearly updates (213). Seventeen sustainable development goals (SDGs) were presented to achieve meaningful improvements by 2030. Ending poverty and other deprivations is advocated while implementing strategies to improve health and education, reduce inequality, spur economic growth, and reduce the adverse effects of climate change. The SDGs require the maintenance of lifelong brain health priorities (214).

Polycrisis conditions such as the recent COVID-19 pandemic and worldwide armed conflicts reaffirm the continued need to prioritize healthcare policies that protect vulnerable women and children exposed to extreme adversities (215). Synergy among disciplines stresses the sustainability of brain health across each lifespan through shared learning of environmental neuroscience relevant to the neural exposome by healthcare providers and stakeholders. A science-based agenda for life course health policy can potentially mitigate TSI effects to preserve brain health for successive generations (216, 217).

Interdisciplinary FNN training promotes person-centered research collaborations between pediatric and adult neurologists. Multi-institutional diagnostic and treatment protocols need to be applicable to all women, children, and families exposed to TSI. Age-sensitive diagnostic modalities using genotypic-phenotypic biomarkers will promote effective neurotherapeutic developments tailored to critical-sensitive time periods of neuroplasticity across the lifespan (218).

## Author contributions

MS: Conceptualization, Formal analysis, Investigation, Writing – original draft.
