# Supplementary material for: Interdisciplinary fetal-neonatal neurology training applies neural exposome perspectives to neurology principles and practice
Source: Front Neurol. 2024 Jan 15;14:1321674. doi: 10.3389/fneur.2023.1321674 (PMC10824035; doi:10.3389/fneur.2023.1321674)
Supplement: Supplementary file 1 [file Data_Sheet_1.pdf]

APPENDIX 1 –NEONATAL NEUROCRITICAL CARE COMPONENT

CONCEPT OVERVIEW

1. Neural exposome-endogenous/exogenous stressor interplay
2. Gene-environment interactions
3. Developmental neuroplasticity
4. Developmental origins of health- disease
5. Life-course theory
6. Social determinants of health
7. Bioethical principles and practice
8. Science of uncertainty
9. Cognitive-based decision making
10. Sources of bias-debiasing strategies

PERIPARTUM/LABOR AND DELIVERY

1. Maternal reportage
2. Antepartum/intrapartum fetal surveillance
3. Resuscitation strategies and testing
4. Stabilization and initiation of interventions

SERIAL EXAMINATIONS-ASSESSMENTS

1. Apgar, Sarnat, Thompson scores
2. Anthropometric measures
3. Vital signs-autonomic nervous system
4. System-specific assessments
5. Specific neurologic exam components
6. Sign and symptom evolution over time
7. System-specific testing tools
8. Pain and stress assessments
9. Developmental assessments
10. Discharge planning with PCP and early intervention programs

MULTI-SYSTEMIC DISORDERS

1. Complex congenital heart disease
2. Pulmonary hypertension
3. Sepsis
4. Fetal inflammatory response syndrome
5..System specific anomalies
6. Necrotizing enterocolitis
7. Hydrops fetalis-immune/non-immune types
8. Hepatobiliary diseases
9. Metabolic/toxic disturbances
10. Neonatal abstinence syndrome
11. Maternal medication/stressor effects

NEURODIAGNOSTIC TOOLS

1. Neuroimaging-cranial sonography, brain CT and MRI studies, MRS, computer imaging choices
2. Neurophysiological studies- continuous (video) EEG, aEEG, EEG trend algorithms, evoked response studies
3. Near-infrared spectroscopy
4. Placental-cord pathology
5. Neuropathology
6. Multisystem autopsy findings

NEONATAL ENCEPHALOPATHY (NE)

1. Antepartum, peripartum, intrapartum timing
2. Etiopathogenetic pathways
3. NE mimicry
4. Encephalopathy of prematurity

NEONATAL SEIZURES

1. Multi-tier classification; recognition, localization, timing, etiopathogenesis
2. Neuromonitoring
3. Paroxysmal disorders
4. Treatment/discontinuation decisions using antiepileptic medications.
5. Risks for epilepsy and co-morbidities
6. Genetic biomarkers for epilepsy risk

CEREBROVASCULAR DISORDERS

1. Incidence-prevalence
2. Arterial-venous developmental origins
3. Etiopathogenetic pathways
4. Neurodiagnostic evaluations
5. Intracranial hemorrhages- full term, preterm
6. Co-morbidities and prognosis

CNS MALDEVELOPMENT

1. Cerebrum – malformations based on disorders of neurogenesis through synaptogenesis
2. Midline lesions: holoprosencephaly, corpus callosum, septum pellucidum defects
3. Posterior fossa structures brainstem/cerebellar midline-hemispheric
4. Anomalous versus destructive lesions
5. Spinal cord development (e.g., neural tube defects, caudal regression)
6. Ventriculomegaly

CENTRAL-PERIPHERAL INJURIES

1. Craniocerebral trauma – extradural, subdural subgaleal hemorrhages,
2. Skull fracture categories
3. Brachial plexus injuries
4. Spinal cord injuries
5. Cranial and peripheral nerve injuries

NEUROGENETICS

1. Chromosomal & genetic evaluations
2. Neurocutaneous disorders
3. Inborn errors of metabolism by pathway
4. Neurodegenerative diseases
5. Posttranslational advances
6. Mosaicism, somatic variation, epigenetics

NEUROMUSCULAR

1. Lower motor neuron pathway development
2. Neonatal hypotonia-hypertonia
3. Arthrogryposis
4. Spinal muscular atrophy
5. Congenital myopathies
6. Critical illness myopathy

NEUROPROTECTION

1. Neuromonitoring devices/procedures
2. Preventive, rescue, reparative choices
3. Pharmacologic interventions
4. Therapeutic hypothermia-EPO
5. Non-pharmacologic interventions
6. Developmental care choices
7. Studies of novel treatment options

PARENTAL-FAMILY INTERACTIONS

1. Medical-based decisions
2. Value-based decisions
3. Shared decision making
4. Bioethical standards
5. Palliative care/end of life decisions
6. Autopsy requests and discussions

APPENDIX 1B –REPRODUCTIVE AND PREGNANCY HEALTH COMPONENTS

PRE-CONCEPTION

1. Adolescent-related pregnancy complications
2. Hypertensive disorders
3. Metabolic/endocrine disorders
4. Obesity and related disorders (PCOS)
5. Neuropsychiatric disorders & medications
6. Epilepsy & antiepileptic medications
7. Chronic neurological disorders (e.g., multiple sclerosis, neuromuscular diseases)
8. Multisystemic genetic disorders
9. Male-specific conditions

FERTILITY ISSUES & TESTING

1. Pre-implantation genetic diagnosis
2. Infertility
3. Artificial reproductive technologies

PRENATAL SCREENING

1. Maternal serum screening
2. First trimester screening
3. Cell free DNA / noninvasive prenatal testing (NIPT)
4. Neonatal Autoimmune Thrombocytopenia (NAIT) testing
5. TORCH testing
6. Carrier testing
7. Thrombophilia
8. Amniocentesis/chorionic villus sampling
9. Sonographic arthrometry, Doppler, Biophysical scales
10. Fetal MRIs
11. Fetal magnetoencephalogram (MEG)

NEURAL DEVELOPMENT

1. Embryonic and fetal structures
2. Range of malformations
3. Neural tube defects
4. Ventriculomegaly
5. Omics approach

GROWTH ISSUES

1. Normative growth curves
2. Fetal growth restriction
3. Microcephaly
4. Oligo-polyhydramnios

FETAL FUNCTIONAL NEURODEVELOPMENT

1. Fetal movements
2. State development/organization
3. Autonomic functions
4. Disease correlates; fetal seizures

PERINATAL PATHOLOGY

1. Developmental anatomy- function, “omics” biomarkers
2. Gross pathology -placenta, cord, uterus
3. Histopathology: chorioamnionitis-funisitis, malperfusion, villitis, villous dysmaturity
4. Neuropathological correlates with placental pathological processes.

CHROMOSOMAL & GENETIC SYNDROMES

1. Trisomies – 21; 13; 18
2. Microarray abnormalities
3. Rapid WES/GWGS testing
4. Epigenetic advances-methylome

VASCULOGENESIS/ANGIOGENESIS

1. Arterial/venous development
2. Intraventricular hemorrhages
3. Intraparenchymal hemorrhages
4. Extracranial hemorrhages
5. Ischemic /hemorrhagic stroke syndromes
6. Vascular malformations

INFECTIONS

1. TORCH
2. HIV
3. Zika virus
4. SARS 2
5. Parvovirus
6. Lymphocytic choriomeningitis virus

NEUROMUSCULAR SYSTEM

1. Clubfoot
2. Arthrogryposis
3. Fetal dyskinesia syndrome
4. Genetic neuromuscular diseases

CONNATAL TUMORS

1. Tuberous sclerosis
2. Neurocutaneous melanosis
3. Germ cell tumors – teratoma
4. Supra-infratentorial tumors

COUNSELLING AND END OF LIFE ISSUES

1. Termination – state & federal laws
2. Still births
3. Postnatal family planning
4. Recurrence risk counseling
5. Comfort or unretaliative care
6. Autopsy requests and genetic testing

FETAL THERAPIES

1. Fetal infections for TORCH,HIV
2. Maternal treatments -autoimmune, metabolic, nutritional disorders
3. Fetal surgery – myelomeningocele; tracheoesophageal fistula; congenital diaphragmatic hernia
4. Vascular – arteriovenous malformations; lymphatic malformations
5. Novel proposed protocols.

APPENDIX 1C–PEDIATRIC COMPONENT (<2 years of age)

OUTPATIENT/INPATIENT SERVICES

1. NICU discharge planning
2. PCP referrals-wellness programs
3. Early intervention services
4. Pediatric subspecialty referrals
5. Medically fragile child
6. Adverse childhood effects
7. Hospitalizations
8. PICU admissions
9. Surgeries

STANDARDIZED ASSESSMENT TOOLS

1. Neonatal
2. Neurobehavioral
3. Gross motor
4. Speech/Language
5. Cognitive testing
6. Social-adaptive testing

HEARING ASSESSMENTS

1. Audiologist evaluations
2. Otoacoustic emissions
3. Brainstem evoked responses

VISION ASSESSMENTS

1. Visual acuity and fields
2. Retinopathy of prematurity
4. Cerebral visual impairment

OUTCOMES ASSESSMENT

1. Vision impaired
2. Hearing impaired
3. Global developmental delay
4. Cerebral palsy
5. Neurobehavior– DSM-TR 5 criteria
6. Cognitive skill testing-executive function
7. Intellectual disability categories
8. DSM-5 classification of mental health Disorders (including TR revisons)

THERAPIES & REHABILITATION

1. Physical therapy
2. Occupational therapy
3. Speech/language therapy
4. Nutrition/feeding therapy
5. Aqua therapy
6. Vision therapy
7. Computer-assisted communication
