# Supplementary material for: Interdisciplinary fetal-neonatal neurology training applies neural exposome perspectives to neurology principles and practice
Source: Front Neurol. 2024 Jan 15;14:1321674. doi: 10.3389/fneur.2023.1321674 (PMC10824035; doi:10.3389/fneur.2023.1321674)
Supplement: Supplementary file 2 [file Data_Sheet_2.pdf]

APPENDIX 2: OUTLINE OF FNN TOPICS TO DEVELOP EXAM QUESTIONS

Topic Headlines		Percentage of questions
I.	Concepts and Diagnostic Approaches	5%
II	Neonatal NeuroCritical Care	45%
III.	Reproductive/pregnancy care.	10%
IV.	Fetal Neurology	25%
IV.	Pediatric follow up Care	15%

I. Concepts and Diagnostic Approaches (see Appendix 1)

1. Developmental origins
2. Life-course theory
3. Cognitive decision making
4. Neural Exposome
5. Social Determinants
6. Cognitive Decision-making

II. Neonatal Neurology

A. Neurodiagnosis

1. Neurologic examination
2. EEG – vEEG, aEEG
3. Cranial ultrasound and Doppler studies
4. CT scan
5. MRI/MRA/MRV
6. MRS
7. NIRS
8. Placental pathology
9. Neurogenetics

B. Brain development

1. Typical development
2. Malformations of the neocortex and hindbrain
3. Spinal malformations (e.g., neural tube defects)
4. Anomalous versus destructive disease pathways

C. Neonatal seizures

1. Classification
2. Etiology
3. Evaluation
4. Therapy
5. Early onset epileptic encephalopathies
6. Risk of postnatal epilepsy

D. Neonatal brain injuries

1. Trauma including epidural, subdural and subgaleal hemorrhages; fractures
2. Preterm injury including GMH, IVH, PVL, WMI, PHVD
    a. Prevention of IVH

b. PHVD – monitoring ventricular size and treatment

3. Term brain injury
   a. Hypoxic ischemic encephalopathy
   b. Intracranial hemorrhage
4. Infections
   a. Congenital
   b. Bacterial
   c. Viral
   d. Protozoan

E. Stroke
1. Incidence
2. Classification
3. Etiology
4. Evaluation
5. Treatment

F. Neurogenetics
1. Chromosomal disorders
2. Neurocutaneous disorders
3. Inborn errors of metabolism
4. Mitochondrial disorders
5. Neuromuscular disorders
6. Neurodegenerative disease
7. Testing tools: WES, GWAS
8. Post-translational genetic advances

G. Neuromuscular
1. Neonatal hypotonia
2. Congenital myopathies
3. Myotonic dystrophy
4. Metabolic myopathies
5. Arthrogryposis – fetal dyskinesia syndromes
6. Evaluations using neurodiagnostic testing

H. Neuroprotection
1. Neuromonitoring applications
   a. NIRS
   b. EEG
2. Therapeutic hypothermia/ erythropoietin
3. IVH prevention bundle
4. Pharmacologic agents for primary/secondary/tertiary forms of neuronal death

I. Systemic disorders (principal examples)
1. Complex congenital heart disease
2. Pulmonary hypertension
3. Sepsis/fetal inflammatory response syndrome
4. Necrotizing enterocolitis

J. Other toxic or traumatic disorders
   1. Toxic stressor interplay-endogenous/exogenous
   2. Pharmacologic exposures including neuropsychiatric medications
   3. Neonatal abstinence syndrome
   4. Neonatal hyperbilirubinemia

K. Pain management and analgesia/anesthesia exposure

II. Fetal neurology

A. Preconception planning
   1. Preimplantation testing for reproductive health or disease
   2. Fertility issues with testing
   3. Artificial reproductive technologies (e.g., IVF)
B. Prenatal screening
   1. Maternal screening
   2. CVS, amniocentesis
   3. Fetal ultrasound & doppler studies
   4. Fetal MRI
C. Abnormalities of the placenta
   1. Anatomy, function
   2. Normal trimester-specific development
   3. Abnormal pathology (e.g. Amsterdam criteria)
D. Fetal growth relative to maternal-placental-fetal triad health and disease
   1. Normative growth curves (e.g., 21st century indices)
   2. IUGR, SGA, ponderal index
   3. Amniotic fluid dynamics: oligo versus polyhydramnios
   4. Brain-placental axis relationships
E. CNS development
   1. Neuroembryology topics
   2. Transient structures, progenitor cell populations, stage of connectivity
   3. Development and functioning of the neurovascular unit.
   4. Malformations of the developing neuroaxis
   5. Ventriculomegaly and other specific structural markers (e.g. absent septum pellucidum)
F. fetal functional neurodevelopment
   1. Fetal swallowing
   2. Prechtl movement scores
   3. Fetal dyskinesia syndrome
G. Fetal seizures
   1. Early infantile epileptic encephalopathies
   2. Genetic/acquired integrated etiologies.
H. Infections
   1. Pathogen specific
   2. Two forms of fetal inflammatory response
I. Maternal conditions
   1. Disease pathway-specific (e.g., Hypertension, diabetes etc.)
   2. Prescribed medication/substance use affecting fetal neurodevelopment

        3. DSM specific neuropsychiatric disorders
        4. Neurology-specific disorders (e.g., epilepsy, MS, etc.)
    J. Early onset neurodegenerative disorders
    K. Brain tumors
    L. End of life issues
    M. Outcomes and disabilities
    N. Fetal therapies
        1. Infections
        2. Autoimmune
        3. Fetal surgery
        4. Exit strategies.

III. Comprehensive Care of the high-risk neonate
    A. Hearing
    B. Vision
    C. Standardized developmental assessments
        1. Domains
        2. Testing instruments
    D. Outcomes & disabilities (childhood to adulthood)
        1. Intellectual disability
        2. Neurobehavioral/psychiatric disorders
        3. Executive function disorder and other specific cognitive deficits
        4. Visual changes including cortical visual impairment (e.g., CVI)
        5. Hearing deficits
        6. Sensory integration disorders
        7. Neurologic disorders associated with reproductive senescence.
    E. Therapies and rehabilitation
        1. Neonatal
        2. Post-discharge; age specific
        3. Shared Clinical Decisions
        4. Neuropalliative Care
